# Mesopores in Metal–Organic Frameworks Rendering Enhanced Rate Capability of Pore-Confined Polyaniline for Supercapacitors

**DOI:** 10.1021/acsami.5c16579

**Published:** 2025-10-07

**Authors:** Tsan-Yu Chuang, Cheng-Hui Shen, Hsin-Ya Tsai, Yu-Chi Wang, Ya-Mei Weng, You-Chi Liang, Cheng-Yan Hsieh, Kuan-Chu Wu, Chi-Lun Chuang, Chung-Wei Kung

**Affiliations:** † Department of Chemical Engineering, 34912 National Cheng Kung University, Tainan City 70101, Taiwan; ‡ Program on Key Materials, Academy of Innovative Semiconductor and Sustainable Manufacturing, National Cheng Kung University, Tainan City 70101, Taiwan

**Keywords:** conducting polymer, hierarchical pores, in-situ polymerization, nanocomposites, pseudocapacitor, zirconium-based MOF

## Abstract

Polyaniline (PANI) has been extensively studied as a pseudocapacitive material for electrochemical energy storage, and the facile mass transfer of counterions in PANI during the electrochemical process is crucial to achieve the excellent rate capability of the corresponding supercapacitor. Nanoporous materials with rigid and interconnected pores are thus considered as attractive scaffolds of PANI to facilitate mass transfer. Herein, soft-template-assisted approaches are employed to synthesize a water-stable zirconium-based metal–organic framework (MOF), UiO-66-NH_2_, with distinct hierarchical pore sizes. Three kinds of UiO-66-NH_2_ nanocrystals with almost the same microporosity and specific surface area, but possessing large mesopores (15 nm), small mesopores (5–6 nm) and no mesopores, respectively, are synthesized. These MOFs are employed as additives during the in situ polymerization of aniline to synthesize a series of MOF-PANI nanocomposites with tunable porosity and electrical conductivity. Electrochemical performances of all MOF-confined PANI are tested in acidic aqueous solutions, where the MOF is chemically stable. PANI confined in the MOF with large mesopores can achieve a specific capacitance of 421 F/g at 0.25 mA/cm^2^, outperforming the pristine PANI (378 F/g). In addition, all the three PANI confined within MOFs exhibit better rate capability compared to the pristine PANI, and the trend of rate capability follows that of the pore sizes. Findings here highlight the importance of the hierarchical porosity and large mesopores in MOFs when MOFs are employed in electrochemical applications, and shed light on utilizing such a mesopore-promoting effect in various MOFs and MOF-based composites for a range of energy-storage and electrocatalytic applications.

## Graphical abstract

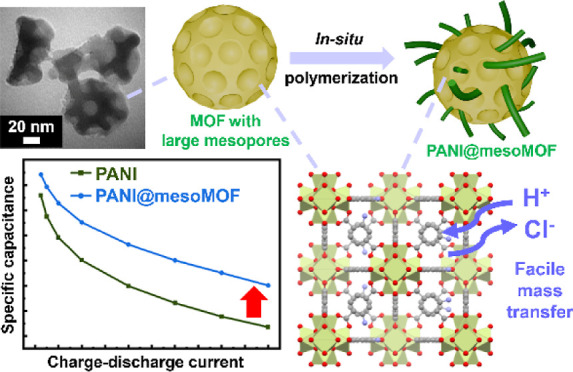

## 1 Introduction

Owing to their unique characteristics, including ultrahigh specific surface areas, regular and interconnected pore structures and tunable chemical functional groups in the pores,^1–4^ metal–organic frameworks (MOFs) have been widely explored for a range of applications.^5–10^ In particular, the rise of tetravalent metal-based MOFs, such as zirconium-based MOFs (Zr-MOFs),^11^ which are exceptionally stable in aqueous solutions ranging from strongly acidic to weakly alkaline pH,^12–14^ has extended the use of MOFs in the applications requiring aqueous media. Thin films of such stable and porous MOFs deposited on electrodes are thus highly attractive for electrochemical energy storage, electrocatalysis and electrochemical sensors, where aqueous electrolytes are usually required.^15–18^ With redox-active moieties in the framework to provide the pathway for charge transport, such a framework can be employed as the support of spatially accessible active sites for electrochemical reactions to occur.^16,19–22^ On the other hand, even with redox-innocent and electrically insulating features, such highly stable and porous MOFs can also be utilized in composites to boost the properties or performances of another electrochemically active material.^15,16,23^

Supercapacitors are fast-charging devices bridging the gap between batteries and conventional capacitors.^24^ Among various active materials for supercapacitors, polyaniline (PANI) has been extensively studied owing to its high theoretical specific capacitance.^24–27^ The charge-storage process of PANI relies on its fast and reversible Faradaic reactions coupled with the adsorption and desorption of counterions;^26,28,29^ such materials are commonly categorized as pseudocapacitive materials.^30,31^ The fast electronic conduction and the facile mass transfer of ions in PANI are thus both crucial in order to achieve a good rate capability, i.e., maintaining high specific capacitances at fast charging–discharging rates.^32^ To achieve this goal, utilizing highly porous MOFs to prepare MOF-PANI composites has become an effective route to prevent PANI from aggregating as well as provide continuous pores for the fast mass transfer of ions.^33–36^ It is worth noticing that to synthesize PANI with a high electrical conductivity, acidic environments are usually required during the polymerization,^37,38^ and the use of acidic aqueous electrolytes is necessary for PANI to achieve high specific capacitances.^24–26^ With chemical stability in acidic solutions, Zr-MOFs are thus attractive candidates for preparing MOF-PANI composites. The in situ polymerization of aniline within the pores of Zr-MOF crystals is feasible to synthesize MOF-PANI nanocomposites while preserving the structural integrity of the MOF.^33^ Such Zr-MOF-PANI nanocomposites have been widely reported in previous studies published by various groups and us,^33,35,36,39–43^ and some of them were applied for supercapacitors to achieve outperforming performances compared to the pristine PANI.^35,36,41,43^ But in most published examples, Zr-MOFs with small micropores, i.e., pores with sizes less than 2 nm, were used to form the MOF-PANI composites. We thus hypothesized that by further expanding the pore sizes of Zr-MOFs to provide more facile mass transfer of ions within the material, PANI in the resulting nanocomposite should achieve a better capacitive performance.

However, creating structurally well-defined pores in Zr-MOFs with sizes in the range of large mesopores is challenging; most Zr-MOFs reported so far still possess pore sizes smaller than 5 nm in their crystallographic structures.^44,45^ On the other hand, by employing the soft-template-assisted synthesis, it is feasible to create ordered large mesopores in Zr-MOF crystals, though these large pores are not from the crystallographic structure of the MOF.^46–51^ For example, by introducing triblock polymers as surfactants to form soft templates during the growth of MOFs followed by the removal of templates, large mesopores up to 40 nm could be created in MOF crystals while fully preserving the microporosity originating from the MOF structure.^52–54^ We thus reasoned that such hierarchically porous Zr-MOFs should be highly appealing for aqueous electrochemical processes to provide facile mass transfer of ions. However, studies on the effect of large mesopores in MOFs on the resulting electrochemical performances are fairly rare in the literature. In our recent work, we first reported that large mesopores in a MOF thin film could facilitate the mass transfer of ionic reactants and thus accelerate the electrochemical reaction occurring on the pore-confined metallic nanoparticles.^55^ In addition, mesopores in a Zr-MOF could also enhance the ion-coupled charge-hopping process between redox-active sites immobilized in the framework, as discovered in another recent study from our group.^56^ But to date, there is not any study reporting the conducting polymers in MOFs with large mesopores and their corresponding electrochemical performances.

Herein, soft-template approaches were employed to synthesize a Zr-MOF, UiO-66-NH_2_, with diverse sizes of large mesopores in the crystals. As illustrated in Figure 1, by utilizing a nonionic surfactant, Pluronic F127 (PEO_106_–PPO_70_–PEO_106_), a triblock copolymer composed of poly­(ethylene oxide) (PEO) and poly­(propylene oxide) (PPO), UiO-66-NH_2_ crystals with mesopores of around 5 nm can be synthesized.^53^ On the other hand, with two kinds of triblock polymers as well as toluene to generate the emulsion in the aqueous solution for MOF growth, large mesopores of around 15–20 nm can be created in UiO-66-NH_2_ crystals.^52^ We then employed these Zr-MOFs with different degrees of hierarchical porosity for the in situ polymerization of aniline to synthesize a series of MOF-PANI nanocomposites. With large mesopores to facilitate the mass transfer of counterions during the charge–discharge process, PANI confined in the mesoporous MOF synthesized from emulsion (E-mUiO-66-NH_2_) can exhibit much better capacitive performance as well as a better rate capability compared to both the pristine PANI and PANI confined in Zr-MOFs with smaller pores.

**Figure 1:**
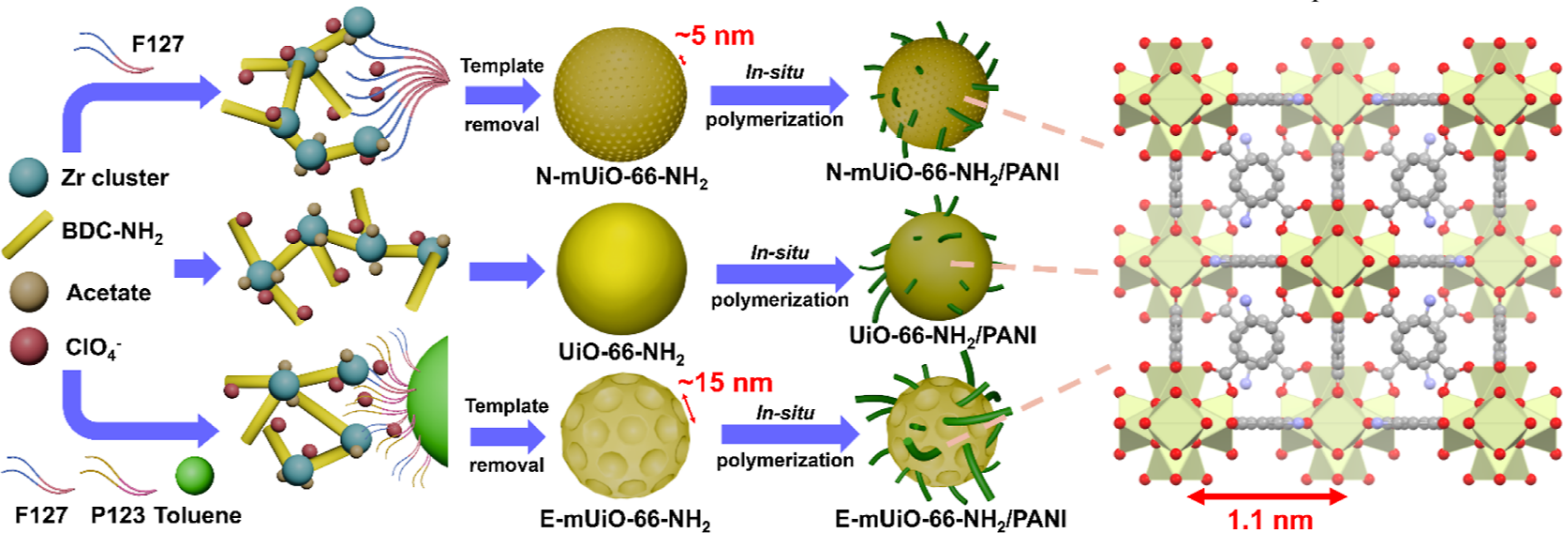
Schematic representation for the aqueous synthesis of UiO-66-NH_2_ nanocrystals, the use of a nonionic surfactant to synthesize UiO-66-NH_2_ nanocrystals with small mesopores, the introduction of emulsion to synthesize UiO-66-NH_2_ nanocrystals with large mesopores, and the corresponding polymerization of aniline in these MOFs. Pale green, red, gray and blue atoms in the MOF structure indicate zirconium, oxygen, carbon and nitrogen, respectively.

## 2 Experimental Section

### 2.1 Chemicals

Zirconium­(IV) oxynitrate hydrate (ZrO­(NO_3_)_2_·*x*H_2_O, 99%), 2-aminoterephthalic acid (H_2_BDC-NH_2_, 99%), pluronic F127 (PEO_106_–PPO_70_–PEO_106_), pluronic P123 (PEO_20_–PPO_70_–PEO_20_), aniline (≥99.5%) and ammonium persulfate (≥98%) were purchased from Sigma-Aldrich. Acetic acid (≥99.8%), nitric acid (HNO_3_, ≥65%), sulfuric acid (H_2_SO_4_, 95.0–98.0%), hydrogen peroxide solution (H_2_O_2_, 30–31%) and potassium bromide (KBr, ≥99.0%) were obtained from Honeywell Fluka. Sodium perchlorate (NaClO_4_, 98.0–102.0%), toluene (99.8%) and *N,N*-dimethylformamide (DMF, 99.9%) were purchased from Alfa Aesar, Tedia and Duksan Pure Chemicals, respectively. Hydrochloric acid (HCl, 36.5–38.0%) was obtained from J. T. Baker. Acetone (>99.0%) and ethanol (95%) were purchased from UNI-ONWARD Corp., Ltd., Taiwan. Deionized water was utilized as the source of water throughout the entire work.

### 2.2 Synthesis of MOFs

To synthesize the UiO-66-NH_2_ nanocrystals with around 5 nm mesopores, F127 was employed as the surfactant during the synthesis; this synthetic procedure has been reported in a previous study.^53^ First, 100 mg of pluronic F127 was dissolved in 6 mL of water in a 10 mL vial by sonication. Thereafter, 0.8 mL of acetic acid and 300 mg of NaClO_4_ were added into the solution, followed by sonicating until a homogeneous mixture was obtained. Subsequently, 231.2 mg of ZrO­(NO_3_)_2_·*x*H_2_O and 100 mg of H_2_BDC-NH_2_ were introduced into the above solution, and the resulting mixture was stirred at 300 rpm in an oil bath at 40 °C for 12 h. The solid product was then collected by centrifugation and washed with 12 mL of water once and 12 mL of DMF three times, sequentially. To remove the surfactant from MOF, the solid was further immersed in 12 mL of ethanol for 4 h, followed by the replacement of the solvent with fresh ethanol for another immersion for 4 h. The solid was then treated with 12 mL of fresh acetone three times to allow the complete solvent exchange, with the immersing periods of 2 h, overnight and 2 h in between. After activating the solid in a vacuum oven at 60 °C overnight, the obtained product was designated as “N-mUiO-66-NH_2_.”

To synthesize the same nanocrystals of the regular UiO-66-NH_2_ without mesopores, the synthetic procedure is exactly the same as that described above, except F127 was not added in the first step.

In order to synthesize UiO-66-NH_2_ nanocrystals with even larger mesopores, nanoemulsion-assisted synthesis with the use of two surfactants and toluene was performed.^52^ First, 200 mg of pluronic P123 and 100 mg of F127 were dissolved in 12 mL of water in a 20 mL vial by ultrasonication until a clear solution was obtained. Thereafter, 320 μL of toluene was added, and the mixture was further sonicated to generate a stable milky emulsion. Afterward, 1.6 mL of acetic acid and 600 mg of NaClO_4_ were introduced, and the mixture was further sonicated. Thereafter, 460 mg of ZrO­(NO_3_)_2_·*x*H_2_O and 200 mg of H_2_BDC-NH_2_ were added into the mixture, and it was stirred at 300 rpm in an oil bath at 40 °C for 12 h. After the growth of MOF, the solid product was subjected to the same washing, solvent-exchange and activating processes described above. The resulting solid was designated as “E-mUiO-66-NH_2_.”

### 2.3 Synthesis of PANI in E-mUiO-66-NH_2_ with Various MOF-to-PANI Ratios

The in situ polymerization of aniline in the presence of MOF was conducted by following a modified procedure reported in our previous work.^36^ The resulting nanocomposites were named as “E-mUiO-66-NH_2_/PANI­(*X*),” where *X* represents the mass ratio between E-mUiO-66-NH_2_ and aniline added during the polymerization. Briefly, 14 μL of aniline, 0.155 mL of concentrated HCl and 3.75 mL of water were mixed in a 20 mL glass vial, and the obtained homogeneous solution was stirred at 1000 rpm for 30 min to acidize the monomer. Subsequently, 3.5, 7, 10.5, or 14 mg of E-mUiO-66-NH_2_, corresponding to *X* = 0.25, 0.5, 0.75 or 1, respectively, was added into the aniline solution along with another 3.75 mL of water. The resulting mixture was further stirred at 1000 rpm for 6 h to allow the complete penetration of aniline into the pores of MOF. Thereafter, 0.04 g of ammonium persulfate, the initiator for polymerization, was dissolved in 2.5 mL of water, and this solution was added into the MOF-aniline mixture dropwise at a rate of 0.25 mL/min under continuous stirring to initiate the polymerization. The mixture was further stirred overnight at 1000 rpm to ensure complete polymerization. The resulting dark green solid was collected by centrifugation and washed with 10 mL of 0.2 M HCl aqueous solutions three times. Thereafter, the solvent-exchange process with acetone was performed, similar to that performed after synthesizing the MOF. Finally, the product was dried in a vacuum oven at 60 °C overnight; E-mUiO-66-NH_2_-based nanocomposites were thus obtained.

### 2.4 Synthesis of PANI in UiO-66-NH_2_, PANI in N-mUiO-66-NH_2_ and the Pristine PANI

As discussed later, the E-mUiO-66-NH_2_/PANI­(0.75) can achieve the best capacitive performance among all nanocomposites. Thus, for comparison, UiO-66-NH_2_/PANI­(0.75) and N-mUiO-66-NH_2_/PANI­(0.75) were prepared by following the identical procedure, except for replacing the E-mUiO-66-NH_2_ with 10.5 mg of UiO-66-NH_2_ or 10.5 mg of N-mUiO-66-NH_2_, respectively. To synthesize the pristine PANI, the same polymerizing procedure was used except no MOF solid was added.

### 2.5 Preparation of Pellets and Modified Electrodes

Each material was pelletized for two-probe current–voltage (*I*–*V*) measurements to determine its electrical conductivity, following the procedure similar to that reported in our previous study.^41^ For preparing modified electrodes, fluorine-doped tin oxide (FTO) glass substrates (7 Ω/sq.) with an exposed area of 0.25 cm^2^ were prepared following the previously established method.^57^ Subsequently, 3 mg of the active material was suspended in 1.25 mL of acetone by sonication to form a homogeneous suspension. Then, 6 μL of the suspension was drop-cast onto the exposed area of the FTO substrate. This drop-casting process was repeated three times to achieve a uniform and complete coverage of the thin film. The mass loading of the active material in every modified electrode prepared in this work is 0.173 mg/cm^2^. Comparable protocols for preparing modified electrodes have been described in our earlier reports.^36,57^

### 2.6 Instrumentation

Scanning electron microscopic (SEM) images were obtained by using a SU-8010 microscope (Hitachi), under an accelerating voltage of 10.0 kV. Transmission electron microscopic (TEM) images were obtained by using a H-7500 (Hitachi), under an accelerating voltage of 60.0 kV. Powder X-ray diffraction (PXRD) and grazing-incidence X-ray diffraction (GIXRD) patterns were collected by using a SmartLab (Rigaku). Nitrogen adsorption–desorption isotherms were collected by a 3Flex (Micromeritics) at 77 K. Prior to every sorption measurement, the sample was degassed at 110 °C overnight using a VacPrep 061 (Micromeritics). Fourier-transform infrared spectroscopy (FTIR) spectra were obtained by employing a Nicolet 6700 spectrometer (Thermo Scientific), and the range for every measurement is 4000–600 cm^–1^. The sample was ground with KBr followed by pelletization prior to FTIR measurements. Inductively coupled plasma–optical emission spectrometry (ICP-OES) analysis was performed on a JY 2000-2 (Horiba Scientific). For ICP-OES quantification, 4.0 mg of MOF or MOF-PANI composite was precisely weighed and filled into a microwave vial, followed by the addition of 0.75 mL of concentrated H_2_SO_4_ and 0.25 mL of H_2_O_2_. The mixture was sonicated until no gas bubbles remained, and the vial was sealed for microwave-assisted digestion at 150 °C for 20 min with the use of an Initiator+ (Biotage). After cooling and depressurizing the mixture, the obtained solution was diluted to 40 mL by adding 3% HNO_3_ aqueous solution prior to the ICP-OES analysis. For ICP-OES analysis, signals of standard solutions containing various concentrations of zirconium at 339 nm were first measured to obtain the calibration curve, and the zirconium concentration of each sample was estimated by averaging the signals at 339 nm from three separate measurements.

Cyclic voltammetric (CV) and galvanostatic charge–discharge (GCD) measurements were performed by using a CHI6273E instrument (CH Instruments). A conventional three-electrode configuration was employed, consisting of an FTO-based working electrode, a platinum foil counter electrode and a Ag/AgCl/NaCl (3 M) reference electrode. All measurements were carried out at room temperature with the use of 20 mL of 1.0 M HCl aqueous solutions as the electrolytes. All CV curves were obtained by scanning from 0 V to +0.8 V and thereafter scanning back to 0 V. The upper and lower potential limits of all GCD measurements are +0.8 and 0 V, respectively.

## 3 Results and Discussion

### 3.1 Characterizations of MOFs with Various Pore Sizes

The morphology of each MOF was first examined by SEM and TEM measurements. SEM images of UiO-66-NH_2_, N-mUiO-66-NH_2_ and E-mUiO-66-NH_2_ are shown in Figure 2a–c, respectively. In addition, their corresponding TEM images are shown in Figure 2d–f. As revealed in Figure 2a,d, UiO-66-NH_2_ is composed of spherical nanocrystals with sizes of around 40–80 nm, and no obvious mesopores can be found within these nanocrystals. On the other hand, with the help of the nonionic surfactant, F127, as the soft template during the MOF growth, the obtained N-mUiO-66-NH_2_ consists of nanocrystals with similar particle sizes and uniformly distributed small mesopores on each particle (Figure 2b). TEM image in Figure 2e further indicates that these mesopores, with sizes of less than 10 nm, are present within each MOF crystal; this morphology is consistent with that of the mesoporous UiO-66-NH_2_ synthesized by the same procedure reported previously.^53^ By further utilizing both F127 and P123 as surfactants and toluene droplets as the swelling agent to form emulsions during the aqueous growth of the MOF, uniformly distributed large mesopores can be observed in nanocrystals of the resulting E-mUiO-66-NH_2_. As revealed in both Figure 2c,f, such mesopores in MOF nanocrystals have sizes of around 15–20 nm, agreeing with those reported previously.^52^

**Figure 2:**
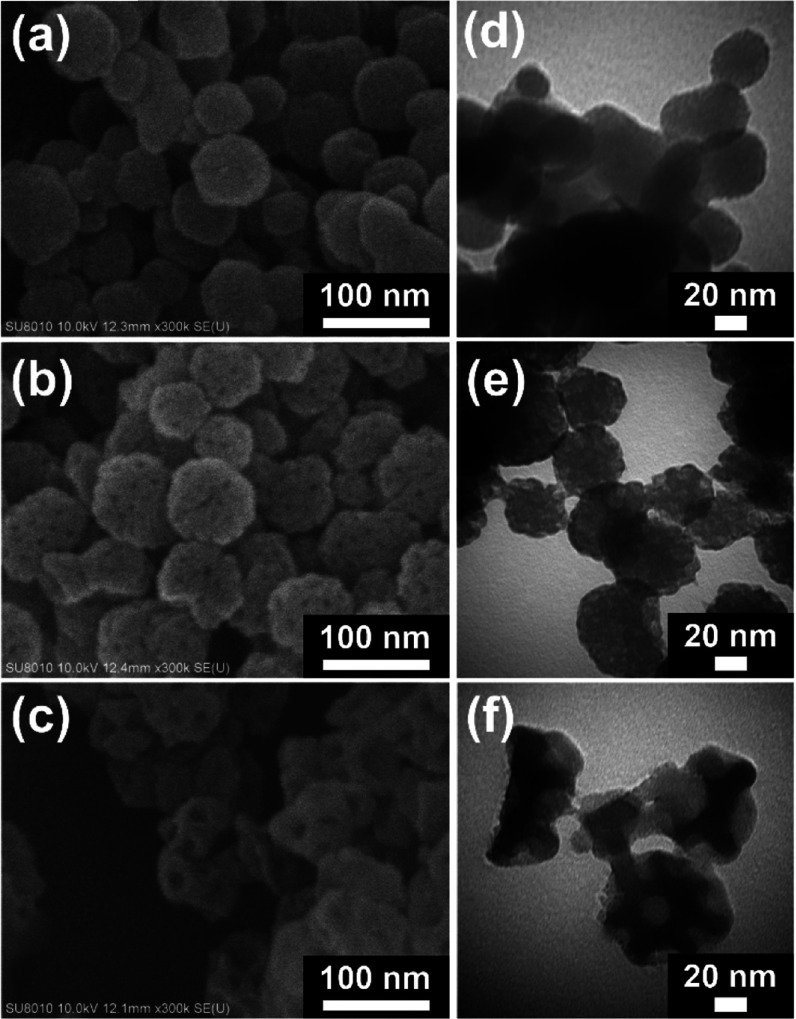
SEM images of (a) UiO-66-NH_2_, (b) N-mUiO-66-NH_2_ and (c) E-mUiO-66-NH_2_. TEM images of (d) UiO-66-NH_2_, (e) N-mUiO-66-NH_2_ and (f) E-mUiO-66-NH_2_.

PXRD patterns of the three MOFs are plotted in Figure 3a. Two major diffraction peaks of UiO-66-NH_2_ located at 7.4 and 8.5° can be clearly found in all the three experimental patterns, indicating that all the three MOFs are composed of well-crystalline UiO-66-NH_2_. It should be noticed that a peak at around 12° can be observed in PXRD patterns of all three materials, which should be a major diffraction peak of UiO-66-NH_2_ as well.^58^ However, this peak in the simulated pattern is too weak to be observable from the plot. A small hump at around 4° can also be seen in the patterns of all the three materials, which should be attributed to the presence of missing-cluster defects in these UiO-66-NH_2_ materials.^59^ Nitrogen adsorption–desorption measurements were then performed to investigate the porosity of each MOF, and the obtained isotherms are shown in Figure 3b. All the three materials possess quite similar isotherms, with one sharp uptake at low relative pressure from structurally well-defined micropores of the MOF and another obvious uptake in the high-pressure region originating from interparticle voids between nanocrystals. Brunauer–Emmett–Teller (BET) surface areas of all the three materials are around 1100 m^2^/g, which agrees well with reported values of UiO-66-NH_2_.^41,56,58^ Pore size distributions of these materials in the region of micropores were first extracted from their isotherms by employing the linear density functional theory (DFT) model.^60,61^ As shown in Figure S1, all the three MOFs possess almost the same pore size distributions in the range of micropores, with the major pore size centered at 1.2 nm originating from the structure of UiO-66-NH_2_ and another size of around 1.5 nm from the defective cavities. Findings here clearly suggest that both the soft-template-assisted procedures for growing MOFs employed here do not significantly affect the structurally derived microporosity of the resulting UiO-66-NH_2_. However, a close look at the isotherms in Figure 3b reveals that the N-mUiO-66-NH_2_ has a small gas uptake with a hysteresis loop in its isotherm at the relative pressure of around 0.4–0.6 (see the inset of Figure 3b), and the E-mUiO-66-NH_2_ possesses an obviously larger uptake in its isotherm in the high-pressure region compared to others. Both observations imply the difference in mesoporosity between these materials. Thus, pore size distributions of these materials in the region of mesopores were further extracted from their isotherms by using the Barrett–Joyner–Halenda (BJH) model.^61,62^ As shown in Figure 3c, all materials possess pore sizes larger than 30 nm, which should be attributed to the interparticle voids as suggested by SEM images. Compared to the UiO-66-NH_2_, which does not have obvious mesopores between 2 and 30 nm, the N-mUiO-66-NH_2_ shows a sharp peak at around 5–6 nm in its pore size distribution, and the pore size distribution of E-mUiO-66-NH_2_ reveals a broad hump centered at around 15 nm. These observations agree well with the mesopores found in SEM and TEM images, which clearly confirm the successful synthesis of two kinds of mesoporous UiO-66-NH_2_ with diverse pore sizes.

**Figure 3:**
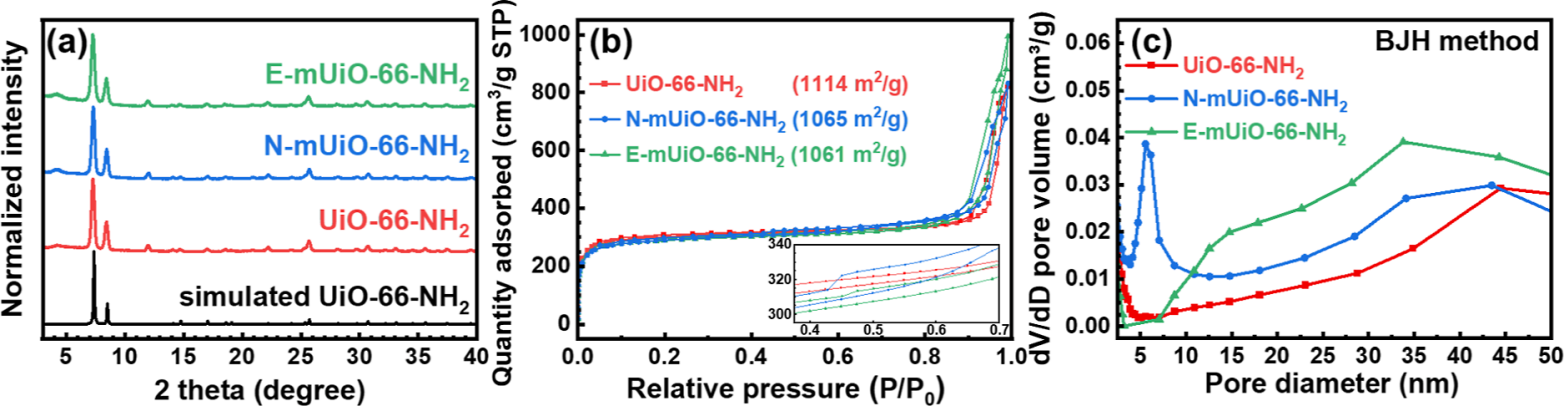
(a) PXRD patterns, (b) nitrogen adsorption–desorption isotherms and (c) BJH pore size distributions of UiO-66-NH_2_, N-mUiO-66-NH_2_ and E-mUiO-66-NH_2_. The simulated PXRD pattern of UiO-66-NH_2_ is shown in (a), and the BET surface area of each sample is listed in (b). Inset of (b) shows the zoom-in version of isotherms.

FTIR measurements were performed to characterize these MOFs as well as to verify the removal of surfactants from these materials. As shown in Figure S2, major characteristic peaks of UiO-66-NH_2_, including those from the stretching vibration of coordinated carboxylate (1656 cm^–1^), bending vibration of N–H (1629 cm^–1^), asymmetric stretching vibration of O=C–O (1574 cm^–1^), vibration of C=C on the aromatic ring (1500 cm^–1^), symmetric stretching vibration of O=C–O (1387 cm^–1^), stretching vibration of C–N (1257 cm^–1^) and wagging of N–H (764 cm^–1^),^56,63^ can be found in spectra of all the three MOFs. On the other hand, both P123 and F127 show strong peaks associated with the stretching vibrations of C–H at around 2900 cm^–1^ in their FTIR spectra.^64^ Such characteristic peaks of surfactants can not be observed in the spectra of all MOF materials, which clearly indicates the complete removal of surfactants from the MOFs during the washing process performed after the MOF growth.

### 3.2 Characterizations of MOF-PANI Nanocomposites

UiO-66-NH_2_, N-mUiO-66-NH_2_ and E-mUiO-66-NH_2_ were thereafter subjected to the in situ polymerization of aniline to synthesize various MOF-PANI nanocomposites; see detailed protocols in the experimental section. It has been reported that during such in situ polymerization, the resulting polyaniline chain could form covalent bonds with amino groups on the linkers of UiO-66-NH_2_, leading to a nanocomposite with strong interactions between the conducting polymer and the MOF host.^43^ As revealed in Figure 4, all the six obtained nanocomposites show diffraction peaks of UiO-66-NH_2_ in their PXRD patterns, which confirms that the structural integrity of the MOF was preserved during the polymerization. The diffraction peaks become obviously weak in the patterns of E-mUiO-66-NH_2_/PANI­(0.25) and E-mUiO-66-NH_2_/PANI­(0.5) since these two materials have lower loadings of MOF compared to others. In particular, the PXRD pattern of E-mUiO-66-NH_2_/PANI­(0.5) shows significantly broadened major peaks of UiO-66-NH_2_ compared to others, which implies that the in situ polymerization of aniline in the presence of a less amount of E-mUiO-66-NH_2_ might reduce the grain sizes of the MOF and thus decrease its crystallinity. On the other hand, the pristine PANI shows an amorphous characteristic in its PXRD pattern with a broad hump located at around 25°.

**Figure 4:**
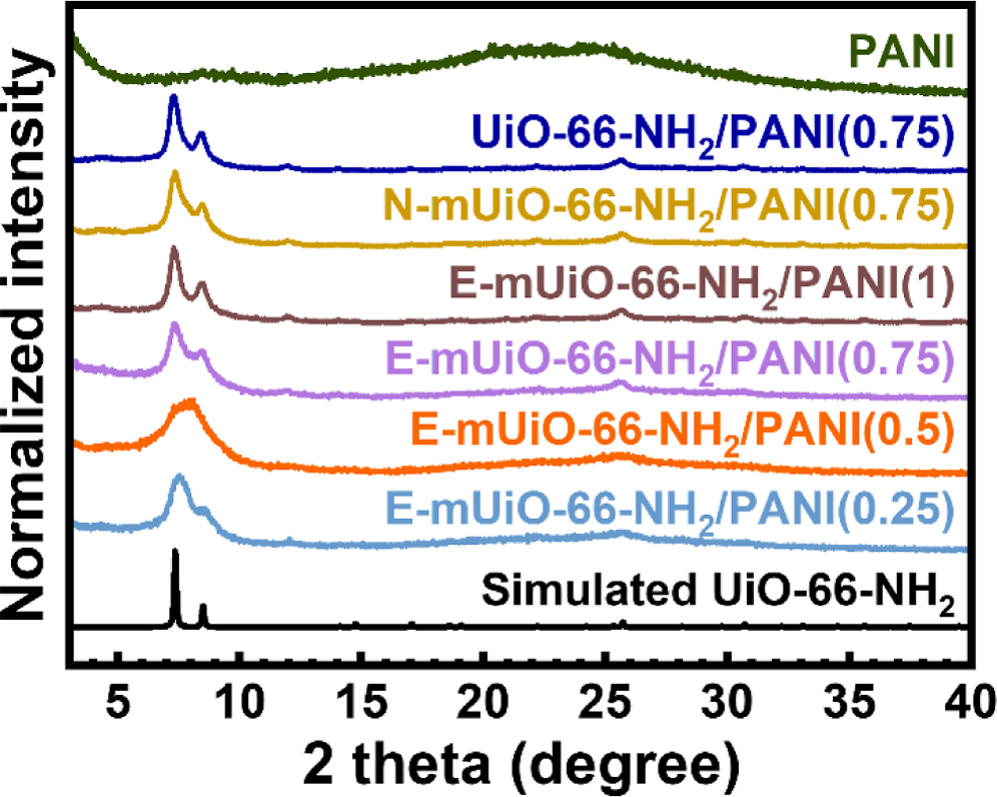
PXRD patterns of PANI and various MOF-PANI nanocomposites.

SEM images of all materials synthesized here were collected in order to probe their morphologies, and the data are shown in Figure S3. The pristine PANI is composed of nanofibers with diameters of around 50 nm (Figure S3­(a)), and these fibers can be found in the images of E-mUiO-66-NH_2_/PANI­(0.25) and E-mUiO-66-NH_2_/PANI­(0.5) as well (Figure S3­(b,c)). However, with increasing amounts of MOF added during the polymerization, the resulting four nanocomposites show particle-like morphologies in their SEM images (Figure S3­(d–g)); fiber-like morphology can not be found in the images of these materials. In addition, mesopores of both E-mUiO-66-NH_2_ and N-mUiO-66-NH_2_ become barely observable on nanoparticles shown in Figure S3­(c–f). Findings here imply that the majority of polyaniline may be confined within the pores of nanocrystalline MOFs in these nanocomposites.

These materials were then subjected to nitrogen adsorption–desorption measurements to examine their porosity, and the obtained isotherms are shown in Figure S4­(a). The pristine PANI is not porous, with a low BET surface area of 51 m^2^/g, and the E-mUiO-66-NH_2_/PANI­(0.25), possessing the lowest loading of MOF among all composites, has almost the same BET surface area as that of PANI. With increasing amounts of MOFs added during the polymerization, the BET surface area of nanocomposite gradually increases. However, even for the E-mUiO-66-NH_2_/PANI­(1), which has more than 60 wt % of MOF inside (see ICP-OES results discussed later), its BET surface area is only 311 m^2^/g, i.e., around 30% of that of the pristine MOF. Results here imply that the solid polyaniline may occupy the major pore volume of MOFs present in these nanocomposites, leading to the remarkable decrease in porosity while preserving the crystallinity of the MOF; the similar observation was also reported for PANI selectively confined in the microporous UiO-66-NH_2_.^41^ DFT and BJH pore size distributions were also extracted from these isotherms, and results are plotted in Figure S4­(b,c), respectively. Compared to the pore size distributions of pristine MOFs shown in Figures S1 and 3c, it can be found that the majority of both micropores and mesopores in all nanocomposites have been occupied by polyaniline.

FTIR spectra of PANI, UiO-66-NH_2_/PANI­(0.75), N-mUiO-66-NH_2_/PANI­(0.75) and E-mUiO-66-NH_2_/PANI­(0.75) are shown in Figure 5, and the spectrum of E-mUiO-66-NH_2_ is also plotted for comparison. In addition to peaks overlapping with those of UiO-66-NH_2_, the pristine PANI has three strong characteristic peaks in its spectrum, associated with the stretching of C–N and C–N^+^ (1299 cm^–1^), bending of C–H from quinoid (1137 cm^–1^) and out-of-plane bending vibration of C–H on the aromatic rings of PANI (802 cm^–1^).^35^ All the three peaks can be clearly observed in spectra of the three nanocomposites shown in Figure 5. On the other hand, major characteristic peaks of UiO-66-NH_2_ can be found in spectra of all nanocomposites as well. Similar characteristics were observed for E-mUiO-66-NH_2_/PANI­(1), E-mUiO-66-NH_2_/PANI­(0.5) and E-mUiO-66-NH_2_/PANI­(0.25) as well, as revealed in Figure S5. Results here indicate that both the polyaniline and UiO-66-NH_2_ are present in all nanocomposites without any obvious change in their chemical structures.

**Figure 5:**
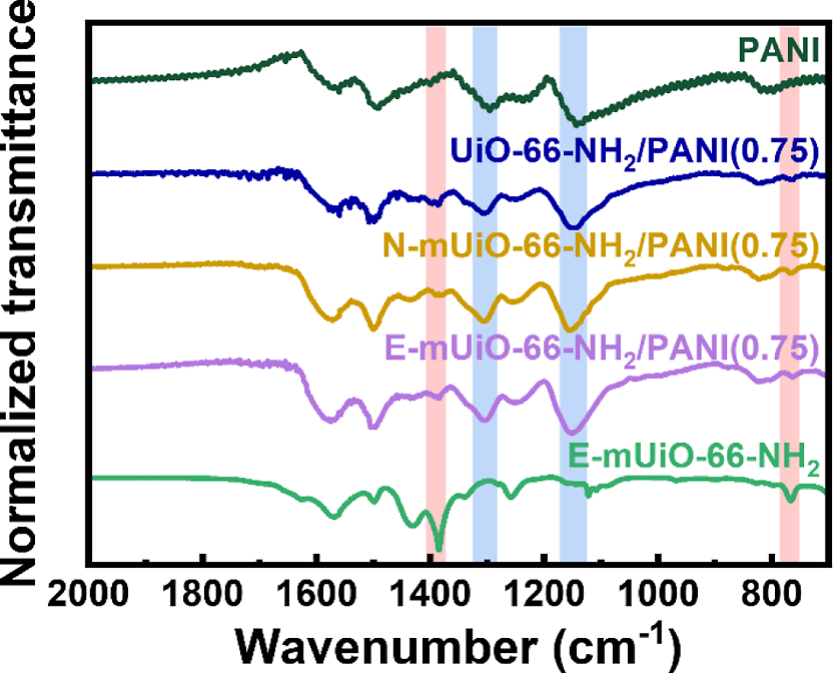
FTIR spectra of PANI, UiO-66-NH_2_/PANI­(0.75), N-mUiO-66-NH_2_/PANI­(0.75), E-mUiO-66-NH_2_/PANI­(0.75) and E-mUiO-66-NH_2_. Selective peaks of PANI and UiO-66-NH_2_ are marked in light blue and light red colors, respectively.

To quantify the mass loading of MOF in each composite material, accurately weighted solid of each material was digested for ICP-OES measurements to quantify its zirconium content. Mass loadings of MOF in all nanocomposites were thus calculated, as listed in Table S1; similar quantifications have been performed for other MOF-PANI composites reported previously.^36,41^ It can be seen that the mass loading of MOF in the resulting nanocomposite increases with the increasing amount of MOF added during the polymerization. In addition, the mass fraction of MOF in each composite is in general higher than the corresponding fraction added, which implies the incomplete polymerization of aniline during the synthesis. Electrical conductivity of each material was thereafter quantified by performing two-probe *I*–*V* measurements; see detailed discussions in the Supporting Information and the protocol in our previous work.^36^ All *I*–*V* curves are shown in Figure S6, and results are summarized in Table S2. It was found that all the three MOFs synthesized here are electrical insulators with electrical conductivity of around 10^–11^ S/cm, and the pristine PANI possesses electrical conductivity of 1.8 × 10^–5^ S/cm. The electrical conductivity, BET surface area, and the mass fraction of PANI calculated from ICP-OES data of E-mUiO-66-NH_2_-based materials are plotted in Figure 6. The porosity of the material decreases significantly with increasing loading of polyaniline. On the other hand, all nanocomposites possess electrical conductivity of around 10^–6^ S/cm. It is worth noticing that the electrical conductivity of E-mUiO-66-NH_2_/PANI­(0.75) (8.7 × 10^–7^ S/cm) is comparable to those of N-mUiO-66-NH_2_/PANI­(0.75) (6.3 × 10^–7^ S/cm) and UiO-66-NH_2_/PANI­(0.75) (7.0 × 10^–7^ S/cm). It suggests that although MOFs in these nanocomposites have quite different pore sizes, the electrical conductivity of the resulting nanocomposites is almost the same.

**Figure 6:**
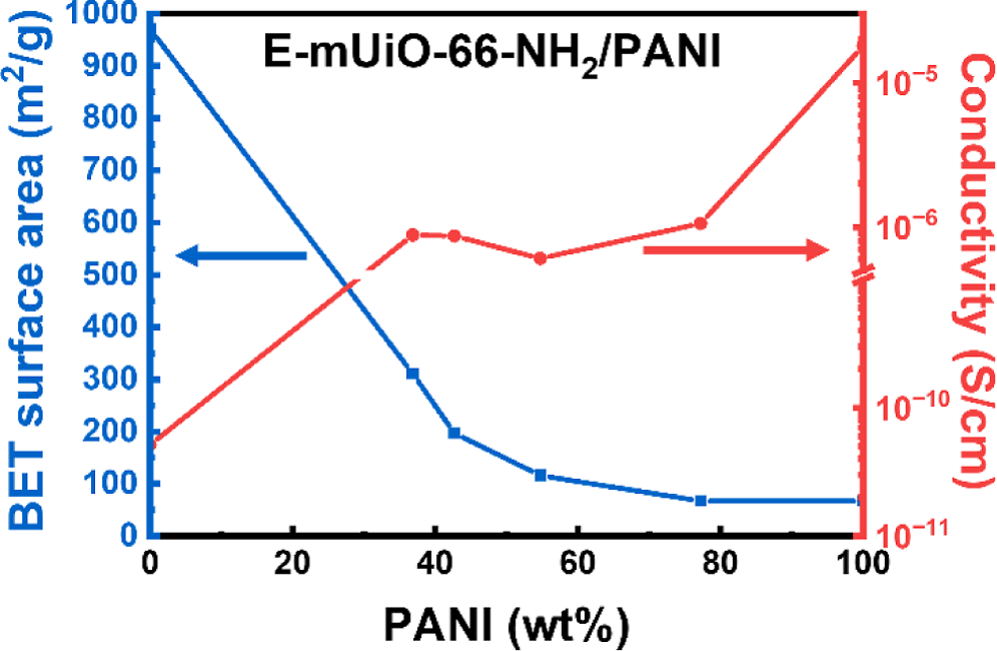
BET specific surface area, electrical conductivity and mass fraction of PANI of all E-mUiO-66-NH_2_-based materials.

It is worth noting that the values of electrical conductivity obtained here are lower than those of PANI typically reported in the literature.^65,66^ The polymerization in this work was performed in an aqueous solution containing around 0.2 M of HCl, and the molar ratio between HCl and aniline is more than ten, maintaining the stably acidic pH during the entire polymerization. However, solvent exchange with acetone was performed for every sample before drying in order to preserve the crystallinity of the Zr-MOF.^67^ This process might be the cause for the relatively low electrical conductivity of all PANI synthesized here, since it has been well-known that the electrical conductivity of PANI highly depends on the final solvent used to dry it.^65^

### 3.3 Capacitive Performances

Given that PANI is widely recognized as a pseudocapacitive material in acidic aqueous electrolytes and UiO-66-NH_2_ is known for its high chemical stability in acids,^24–26,58^ modified electrodes of all materials here were subjected to electrochemical measurements in HCl-based aqueous electrolytes. Prior to further electrochemical tests, we first investigated the structural stability of the three MOFs with diverse pore sizes here in their corresponding nanocomposites during the electrochemical operation. The modified electrode of E-mUiO-66-NH_2_/PANI­(0.75), N-mUiO-66-NH_2_/PANI­(0.75) or UiO-66-NH_2_/PANI­(0.75) was subjected to the CV experiment in the 1.0 M HCl aqueous electrolyte for 50 cycles between 0 and +0.8 V at a scan rate of 50 mV/s. Thereafter, the tested electrode was rinsed with water several times and subjected to the solvent-exchange process with acetone over the course of overnight. GIXRD pattern of each tested thin film after drying was then collected and compared with the corresponding pattern of the freshly prepared thin film. As revealed in Figure S7, the crystallinity of MOFs in all the three thin films can be preserved after electrochemically cycling in 1.0 M of HCl. Aqueous solutions containing 1.0 M of HCl were thus selected as electrolytes for all the following electrochemical experiments.

CV curves of modified electrodes with PANI, E-mUiO-66-NH_2_, N-mUiO-66-NH_2_ and UiO-66-NH_2_ are plotted in Figure S8. Since UiO-66-NH_2_ is electrically insulating and redox innocent within this potential window,^41^ electrodes with all the three pristine MOFs only show small non-Faradaic responses from the underlying FTO electrode in the magnitude of 10^–7^ A/cm^2^; capacitive contributions from these three MOFs are completely negligible compared to that from PANI. Thus, to perform a fair comparison between the capacitive performances and behaviors of the pristine PANI and polyaniline in all nanocomposites, all current responses in CV data of PANI-based electrodes were normalized by the mass loadings of PANI present in corresponding modified electrodes (g_PANI_) according to Table S3.^36^ CV curves of electrodes with E-mUiO-66-NH_2_/PANI­(0.75), N-mUiO-66-NH_2_/PANI­(0.75), UiO-66-NH_2_/PANI­(0.75) and the pristine PANI are shown in Figure 7a. Two sets of reversible and broad redox peaks, centered at around +0.20 V and +0.48 V, respectively, can be found in all CV curves, consistent with the characteristics of PANI reported in the literature.^26,36,66^ These peaks originate from the reversible redox transitions between leucoemeraldine, emeraldine and pernigraniline states of PANI that involve the interconversion of benzenoid and quinoid units.^26,36,66^ CV curves of all E-mUiO-66-NH_2_-based nanocomposites are plotted in Figure 7b, which also reveal the same electrochemical characteristics of PANI. From both Figures, it can be observed that the electrode with E-mUiO-66-NH_2_/PANI­(0.75) exhibits the largest enclosed area in its CV curve among all electrodes, which implies that the PANI confined within E-mUiO-66-NH_2_/PANI­(0.75) should have a slightly higher specific capacitance at this specific scan rate (25 mV/s).

**Figure 7:**
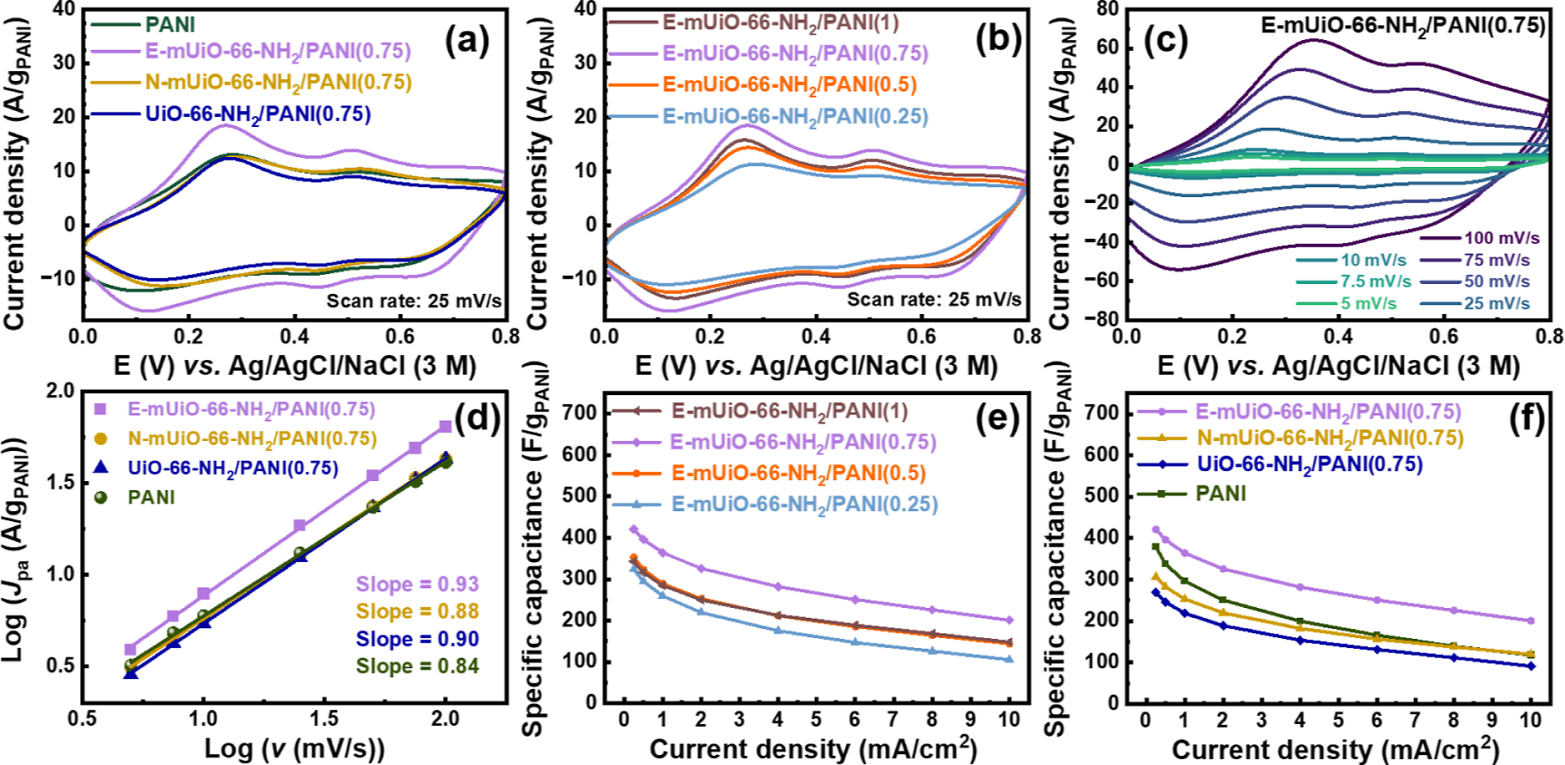
CV curves of modified electrodes with (a) PANI, E-mUiO-66-NH_2_/PANI­(0.75), N-mUiO-66-NH_2_/PANI­(0.75) and UiO-66-NH_2_/PANI­(0.75), and (b) various E-mUiO-66-NH_2_-based nanocomposites, collected at a scan rate of 25 mV/s. (c) CV curves of the electrode with E-mUiO-66-NH_2_/PANI­(0.75) collected at various scan rates. (d) Plot of log­(*J*_pa_) versus log­(*v*), extracted from the data in (c) and Figure S9. Specific capacitance of PANI in (e) E-mUiO-66-NH_2_-based nanocomposites, and (f) PANI, E-mUiO-66-NH_2_/PANI­(0.75), N-mUiO-66-NH_2_/PANI­(0.75) and UiO-66-NH_2_/PANI­(0.75), measured at various charge–discharge rates. Aqueous solutions containing 1.0 M of HCl were employed as electrolytes for all electrochemical measurements.

To further probe the electrochemical kinetics of PANI confined within MOFs with different pore sizes, CV measurements at various scan rates (*v*) were performed for modified electrodes of E-mUiO-66-NH_2_/PANI­(0.75), N-mUiO-66-NH_2_/PANI­(0.75), UiO-66-NH_2_/PANI­(0.75) and the pristine PANI. All obtained CV data are shown in Figures 7c and S9. Two pairs of redox peaks can be clearly observed in all these CV curves, but it is worth noticing that with the pristine PANI, the redox peaks become less well-defined with larger peak separations compared to those of other materials (see Figure S9­(a)); it implies that the pristine PANI may have slightly slower redox kinetics compared to those PANI confined in the three MOFs. Values of anodic peak current density (*J*_pa_) were then extracted from the first anodic peak located between +0.2 V and +0.4 V in each CV curve, and values of log­(*J*_pa_) were plotted with log­(*v*) to obtain the linear relationship, as shown in Figure 7d. A slope of 0.5 in such plots indicates a diffusion-controlled Faradaic process, while a slope of 1.0 suggests that the electrochemical reaction of multiple active sites in the thin film exhibits facile kinetics and behaves similarly to the surface-mounted monolayered redox-active sites.^36,68,69^ As revealed in Figure 7d, the modified electrode with the pristine PANI has a slope of 0.84, indicating that the feature of its Faradaic process is between the diffusion-controlled and surface-controlled behaviors. The slopes become 0.90, 0.88, and 0.93 for UiO-66-NH_2_/PANI­(0.75), N-mUiO-66-NH_2_/PANI­(0.75) and E-mUiO-66-NH_2_/PANI­(0.75), respectively, much closer to 1.0 compared to the slope for the pristine PANI. Results here again indicate that with PANI confined within MOFs in the three nanocomposites, the pore-confined PANI can achieve faster redox kinetics under the electrochemical operation and thus behave more similarly to the surface-mounted monolayered active sites; such a characteristic becomes the most obvious for E-mUiO-66-NH_2_/PANI­(0.75). It should be noted that since all the three nanocomposites tested here have almost the same values of electrical conductivity, and the electrical conductivity of pristine PANI is even 1 order of magnitude higher than those of the three nanocomposites, electronic conduction within these materials should not be the cause for such a difference in redox kinetics. Therefore, such enhanced redox kinetics should be attributed to the faster mass transfer of counterions, i.e., protons and chloride ions here, within the PANI-based thin films. With the rigid MOF scaffold to disperse the PANI in its pores, mass transfer of ions between electrochemically active sites of PANI should be facilitated; such an effect should be more significant when the E-mUiO-66-NH_2_, which possesses the largest mesopores, is present in the composite thin film.

GCD experiments were thereafter conducted to quantify the capacitive performances of PANI at various charge–discharge rates. GCD curves of all modified electrodes are shown in Figure S10. The specific capacitance of PANI in each material (*C*) was thus calculated by using the following equation^36^

1 $$C=\frac{J\times t_{d}}{V\times m}$$

where *J* is the charge–discharge current density, *t*_d_ is the discharging time from the GCD curve, *V* is the potential window, which is 0.8 V here, and *m* is the mass loading of PANI in the thin film from Table S3. Obtained results are plotted in Figure 7e,f. From Figure 7e, it can be seen that the PANI in E-mUiO-66-NH_2_/PANI­(0.75) can exhibit the best capacitive performance among those in all E-mUiO-66-NH_2_-based nanocomposites. Thus, the capacitive performance of E-mUiO-66-NH_2_/PANI­(0.75) was further compared with those of N-mUiO-66-NH_2_/PANI­(0.75), UiO-66-NH_2_/PANI­(0.75) and the pristine PANI, as plotted in Figure 7f. It can be clearly seen that the PANI in E-mUiO-66-NH_2_/PANI­(0.75) exhibits an outperforming capacitive performance compared to others. At a slow charge–discharge rate of 0.25 mA/cm^2^, the PANI in E-mUiO-66-NH_2_/PANI­(0.75) can achieve a capacitance of 421 F/g, obviously higher than those of the pristine PANI (378 F/g), PANI in N-mUiO-66-NH_2_/PANI­(0.75) (308 F/g) and PANI in UiO-66-NH_2_/PANI­(0.75) (270 F/g). Although the enhancement in the capacitance of PANI by employing E-mUiO-66-NH_2_ is not quite obvious at 0.25 mA/cm^2^, the difference becomes much more significant at fast charge–discharge rates. For example, at a fast charge–discharge current of 10 mA/cm^2^, the PANI in E-mUiO-66-NH_2_/PANI­(0.75) can retain a capacitance of 202 F/g, which is 48% of that achieved at 0.25 mA/cm^2^. On the other hand, at 10 mA/cm^2^, the pristine PANI can only retain 31% of its capacitance achieved at 0.25 mA/cm^2^. For N-mUiO-66-NH_2_/PANI­(0.75) and UiO-66-NH_2_/PANI­(0.75), these values are 40% and 34%, respectively. Results here show that the rate capability of PANI follows the trend: E-mUiO-66-NH_2_/PANI­(0.75) > N-mUiO-66-NH_2_/PANI­(0.75) > UiO-66-NH_2_/PANI­(0.75) > PANI, which clearly suggests the effect of all the three MOFs here on boosting the rate capability of PANI. With larger mesopores in the MOF to facilitate the mass transfer of ions, the resulting pore-confined PANI can achieve a better rate capability during the charge–discharge process.

Long-term GCD experiments were conducted for 2000 cycles at a charge–discharge current of 2 mA/cm^2^, and as revealed in Figure S11, the PANI in E-mUiO-66-NH_2_/PANI­(0.75) exhibits almost the same long-term stability compared to that of the pristine PANI. Although the main function of the E-mUiO-66-NH_2_ with large mesopores here is to boost the rate capability of its pore-confined PANI, the optimal specific capacitance achieved here, at a slow charge–discharge rate, was also compared with those of PANI reported in the literature. As shown in Table S4, the specific capacitance achieved here is higher than most reported values of PANI in previous studies.

## 4 Conclusions

Nanocrystals of a Zr-MOF, UiO-66-NH_2_, with diverse hierarchical pore sizes, were successfully synthesized by soft-template-assisted methods. All the three MOFs possess almost the same microporosity originating from the structure of UiO-66-NH_2_, while the E-mUiO-66-NH_2_ and N-mUiO-66-NH_2_ have mesopores of around 15 nm and 5–6 nm in their nanocrystals, respectively. These MOFs can be employed as additives for the in situ polymerization of aniline to generate PANI confined in the pores of MOFs while preserving the crystallinity of them. By adjusting the amount of E-mUiO-66-NH_2_ added during the polymerization, the porosity, electrical conductivity and MOF-to-polymer ratio of the resulting nanocomposite are tunable. Although all MOFs are electrochemically inactive, PANI confined in these MOFs can exhibit reversible electrochemical activity in the aqueous electrolyte containing 1.0 M of HCl, and the crystallinity of the MOF can be well-preserved after electrochemical cycling. With the E-mUiO-66-NH_2_, the MOF with large mesopores, the resulting pore-confined PANI can achieve a higher specific capacitance as well as a much better rate capability compared to the pristine PANI. It was found that the rate capability follows the trend of E-mUiO-66-NH_2_/PANI­(0.75) > N-mUiO-66-NH_2_/PANI­(0.75) > UiO-66-NH_2_/PANI­(0.75) > PANI, which clearly suggests that larger mesopores in the MOF additive can more effectively boost the rate performance of the MOF-confined PANI.

Findings here indicate the importance of hierarchical porosity and large mesopores of MOFs on the resulting electrochemical capacitive performances, even though the MOFs are not electrochemically active and are employed as additives to confine another active material, i.e., PANI. Since water-stable Zr-MOFs have been widely utilized in a range of aqueous electrochemical applications and can play different roles when their crystals are located at the electrode/electrolyte interface,^15–18^ such a promoting effect from large mesopores should be generalizable to other electrochemical processes for both energy and environmental-related applications.

## Supplementary Material



## Notes

DFT pore size distributions, additional FTIR spectra, SEM images and N_2_ gas adsorption and desorption data of nanocomposites, ICP-OES data, *I*–*V* curves and corresponding results of electrical conductivity, GIXRD patterns, additional CV data, GCD curves and long-term electrochemical data (PDF)
