# Supplementary material for: Mesopores in Metal–Organic Frameworks Rendering Enhanced Rate Capability of Pore-Confined Polyaniline for Supercapacitors
Source: ACS Appl Mater Interfaces. 2025 Oct 7;17(42):58314–24. doi: 10.1021/acsami.5c16579 (PMC12557208; doi:10.1021/acsami.5c16579)
Supplement: Supplementary file 1 [file am5c16579_si_001.pdf]

Supporting Information

Mesopores in Metal–Organic Frameworks Rendering Enhanced Rate Capability of Pore-Confined Polyaniline for Supercapacitors

Tsan-Yu Chuang,[a] Cheng-Hui Shen,[a] Hsin-Ya Tsai,[a] Yu-Chi Wang,[a] Ya-Mei Weng,[a] You-Chi Liang,[a] Cheng-Yan Hsieh,[a] Kuan-Chu Wu,[a] Chi-Lun Chuang,[a] and Chung-Wei Kung [a,b*]

[a] Department of Chemical Engineering, National Cheng Kung University, Tainan City, Taiwan, 70101.

[b] Program on Key Materials, Academy of Innovative Semiconductor and Sustainable Manufacturing, National Cheng Kung University, Tainan City, Taiwan, 70101.

* Corresponding author: cwkung@mail.ncku.edu.tw

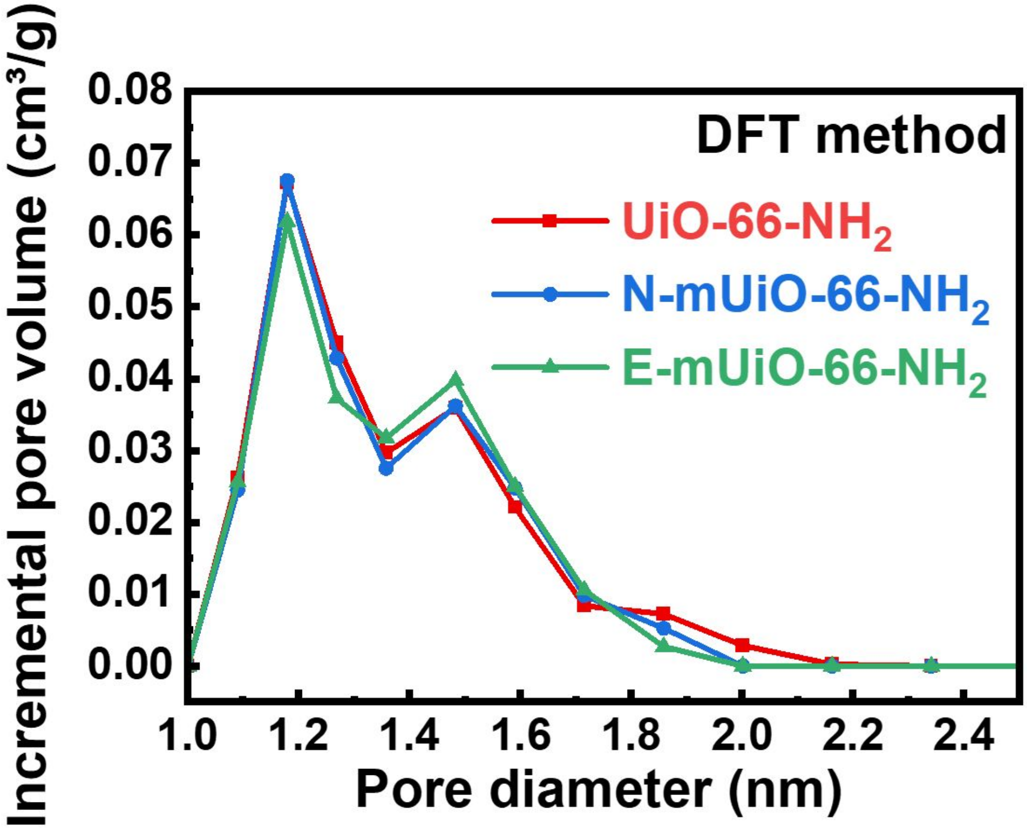

Figure S1. DFT pores size distributions of UiO-66-NH_2, N-mUiO-66-NH_2 and E-mUiO-66-NH_2, obtained from their isotherms shown in the main text.

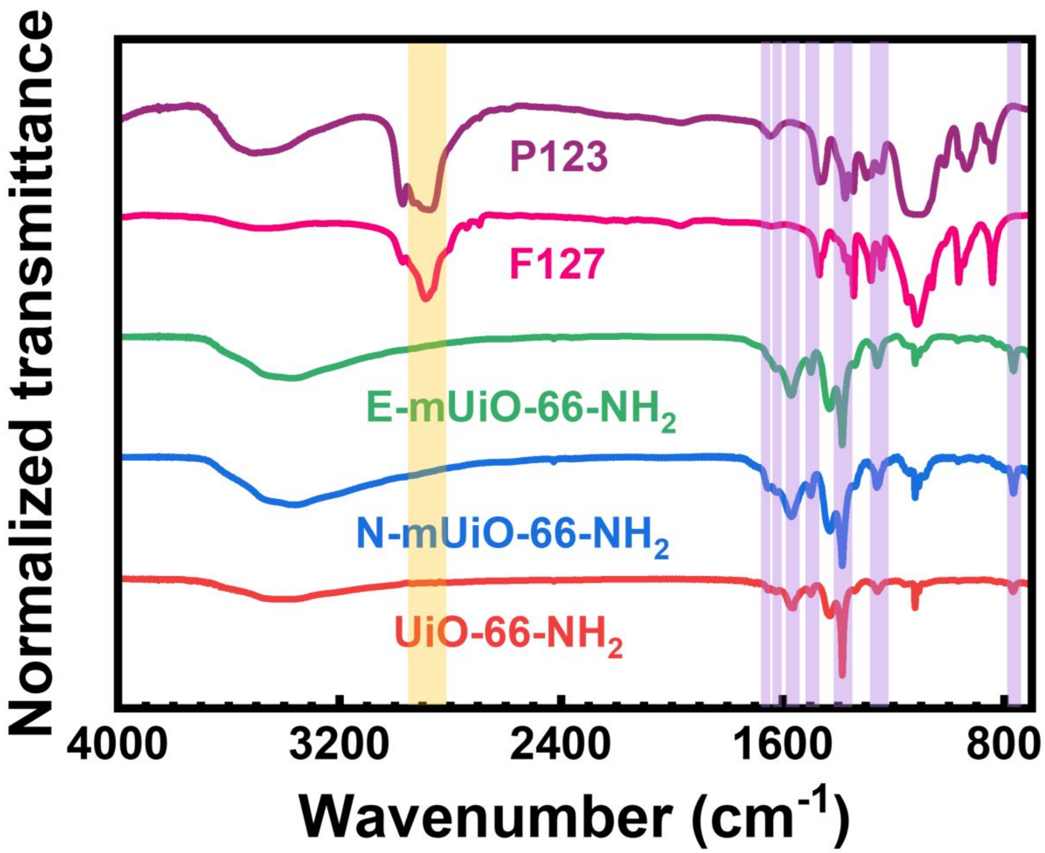

Figure S2. FTIR spectra of P123, F127, E-mUiO-66-NH_2, N-mUiO-66-NH_2 and UiO-66-NH_2. Peaks of MOFs and surfactants are marked in purple and orange colors, respectively.

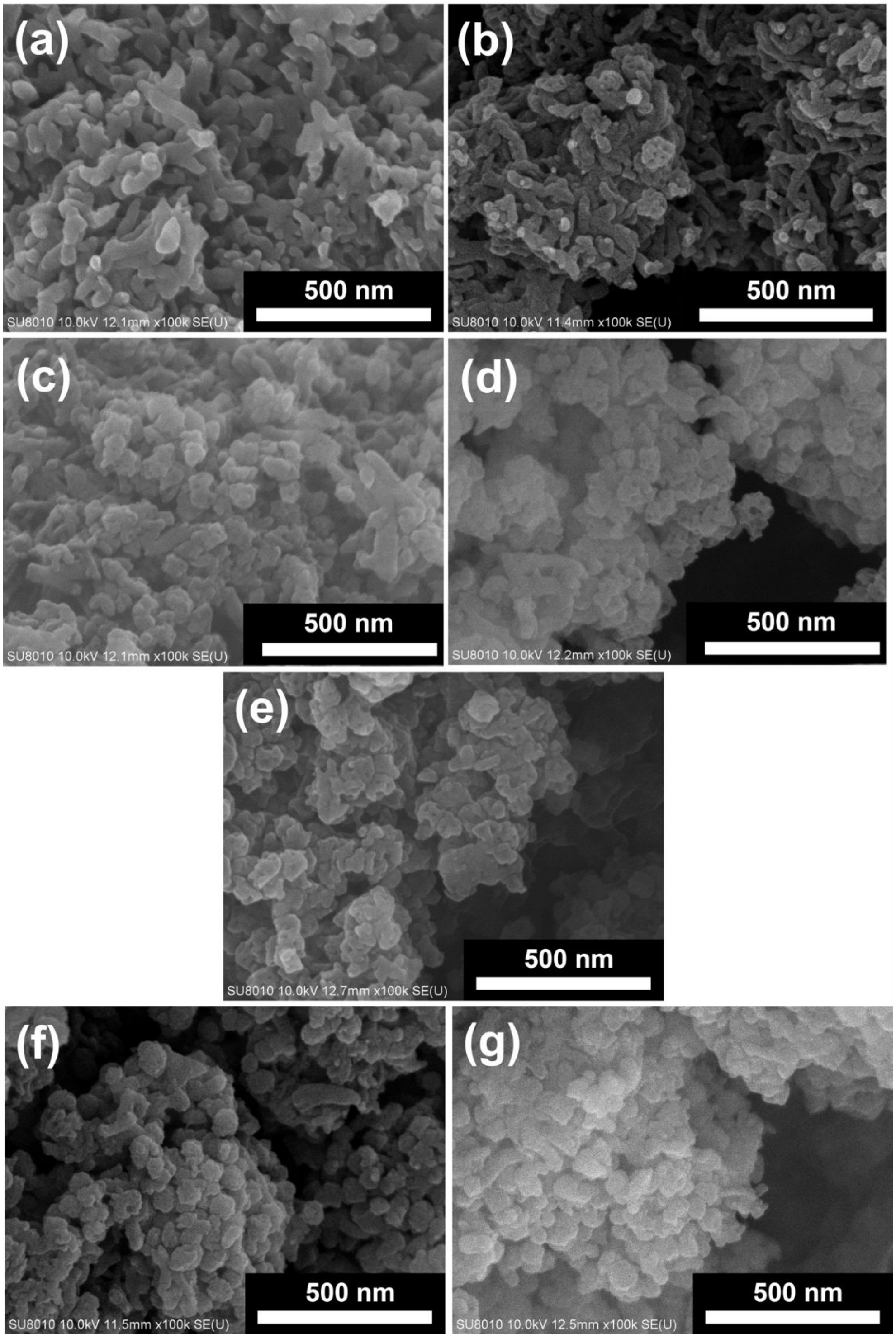

Figure S3. SEM images of (a) PANI, (b) E-mUiO-66-NH_2/PANI(0.25), (c) E-mUiO-66-NH_2/PANI(0.5), (d) E-mUiO-66-NH_2/PANI(0.75), (e) E-mUiO-66-NH_2/PANI(1), (f) N-mUiO-66-NH_2/PANI(0.75) and (g) UiO-66-NH_2/PANI(0.75).

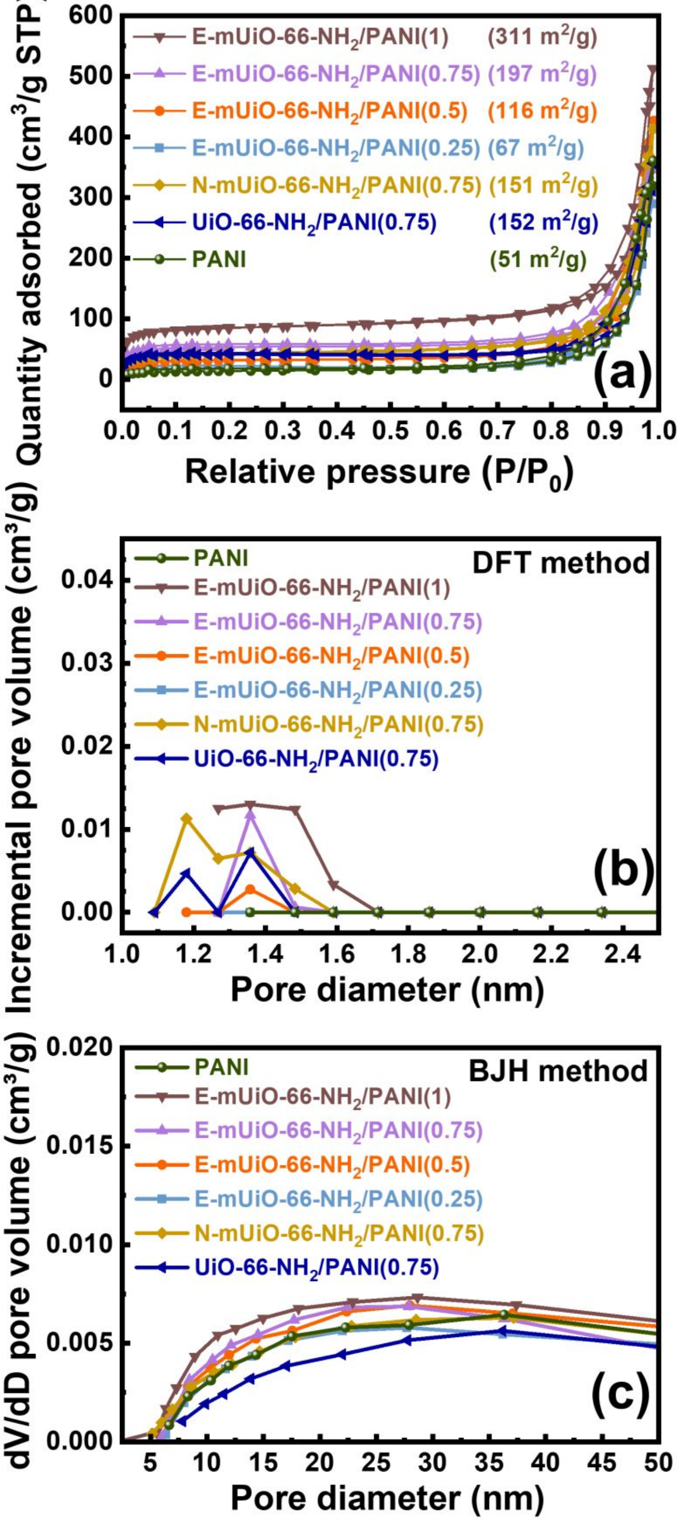

Figure S4. (a) Nitrogen adsorption−desorption isotherms, (b) DFT pore size distributions and (c) BJH pore size distributions of PANI and various MOF-PANI nanocomposites. BET surface areas are listed in (a).

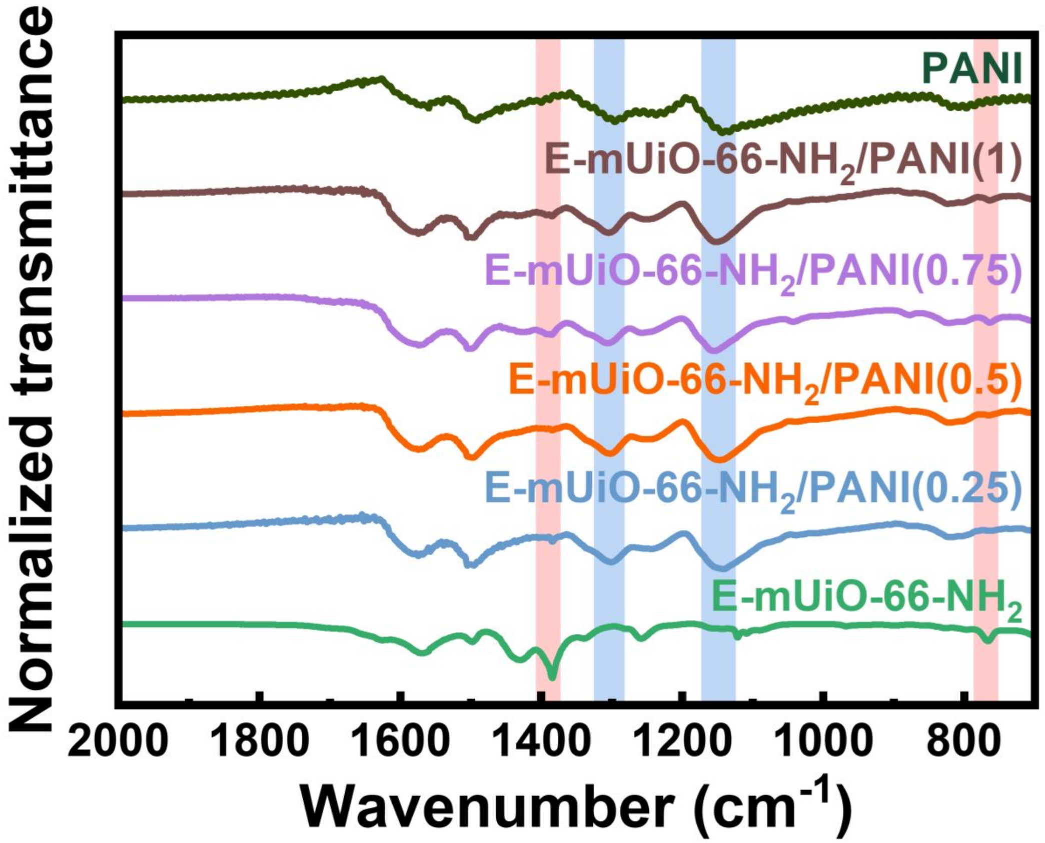

Figure S5. FTIR spectra of PANI, E-mUiO-66-NH_2/PANI(1), E-mUiO-66-NH_2/PANI(0.75), E-mUiO-66-NH_2/PANI(0.5), E-mUiO-66-NH_2/PANI(0.25) and E-mUiO-66-NH_2. Selective peaks of PANI and UiO-66-NH_2 are indicated in light blue and light red colors, respectively.

Table S1. ICP-OES data and the mass fraction of MOF in each material calculated from the ICP-OES results. The mass fraction of MOF added during the synthesis is also listed for comparison.

Materials	Zirconium in ICP-OES sample (ppm)	MOF in final material (wt%)	MOF/(MOF+aniline) added during polymerization (wt%)
E-mUiO-66-NH_2	23.12	100	-
E-mUiO-66-NH_2/PANI(0.25)	5.27	22.8	20.0
E-mUiO-66-NH_2/PANI(0.5)	10.47	45.3	33.3
E-mUiO-66-NH_2/PANI(0.75)	13.25	57.3	42.9
E-mUiO-66-NH_2/PANI(1)	14.6	63.1	50.0
N-mUiO-66-NH_2	22.7	100	-
N-mUiO-66-NH_2/PANI(0.75)	12.61	55.6	42.9
UiO-66-NH_2	24.3	100	-
UiO-66-NH_2/PANI(0.75)	12.58	51.7	42.9

I-V curves of pellets were measured by the two-probe method, and the obtained data are shown in Figure S6. From the slope of each I-V curve, the resistance (R) of each pellet was estimated. The electrical conductivity (σ) of each material was thus calculated by using the thickness of the pellet (L) and the cross-sectional area of the pellet (A). The detailed experimental and calculating methods can be found in our previous studies.[1-4] Obtained results here are listed in Table S2.

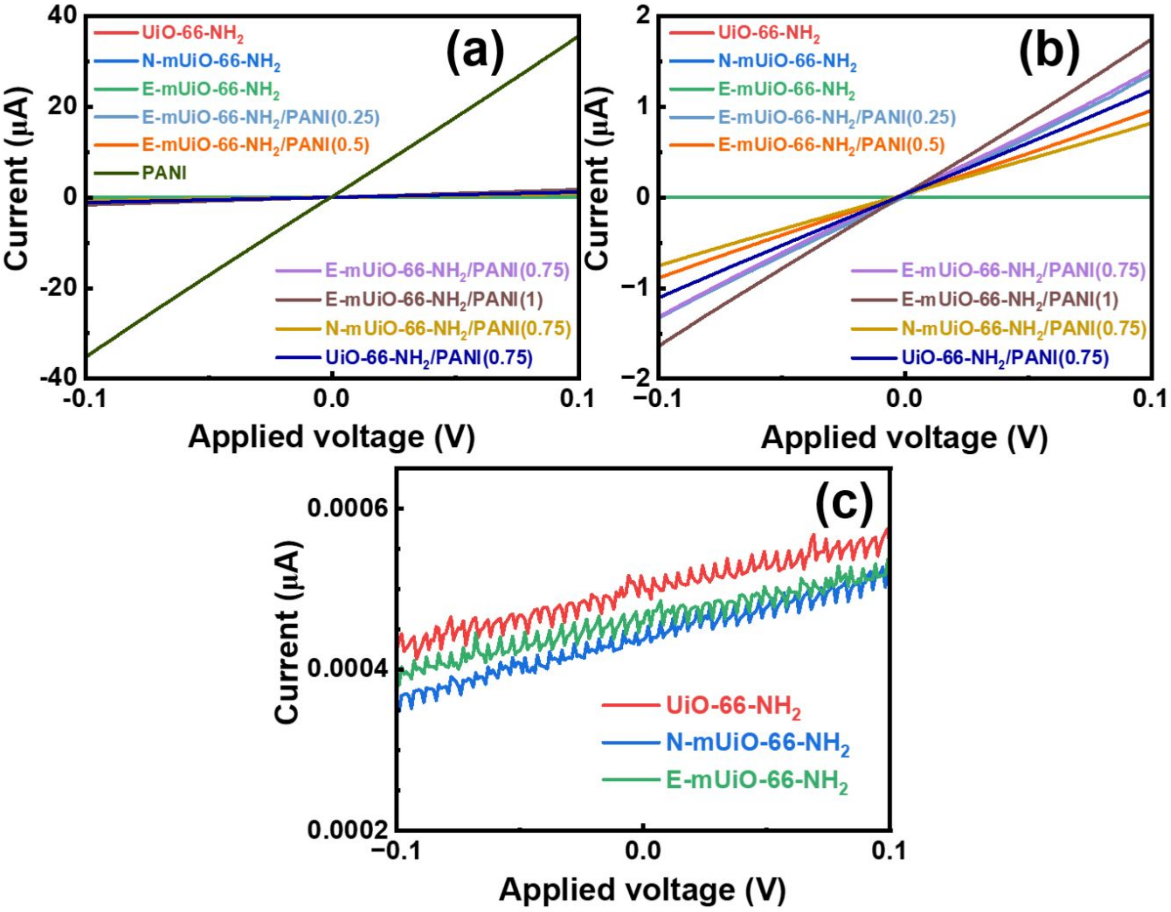

Figure S6. (a) I–V curves of pellets of various materials, measured at a scan rate of 25 mV/s. Zoom-in I-V data, showing curves of (b) the six nanocomposites and (c) the three pristine MOFs.

Table S2. Summary of resistance, thickness, cross-sectional area and calculated electrical conductivity for each pelletized material.

Pellets	R (Ω)	L (cm)	A (cm^2)	σ (S/cm)
UiO-66-NH_2	1.1×10^9	0.023	0.3848	5.7×10^{-11}
N-mUiO-66-NH_2	1.0×10^9	0.031	0.3848	7.8×10^{-11}
E-mUiO-66-NH_2	1.1×10^9	0.022	0.3848	5.2×10^{-11}
E-mUiO-66-NH_2/PANI(0.25)	5.7×10^4	0.023	0.3848	8.3×10^{-7}
E-mUiO-66-NH_2/PANI(0.5)	9.9×10^4	0.023	0.3848	6.1×10^{-7}
E-mUiO-66-NH_2/PANI(0.75)	6.9×10^4	0.023	0.3848	8.7×10^{-7}
E-mUiO-66-NH_2/PANI(1)	5.9×10^4	0.02	0.3848	8.8×10^{-7}
N-mUiO-66-NH_2/PANI(0.75)	1.2×10^5	0.03	0.3848	6.3×10^{-7}
UiO-66-NH_2/PANI(0.75)	8.5×10^4	0.023	0.3848	7.0×10^{-7}
PANI	2.7×10^3	0.019	0.3848	1.8×10^{-5}

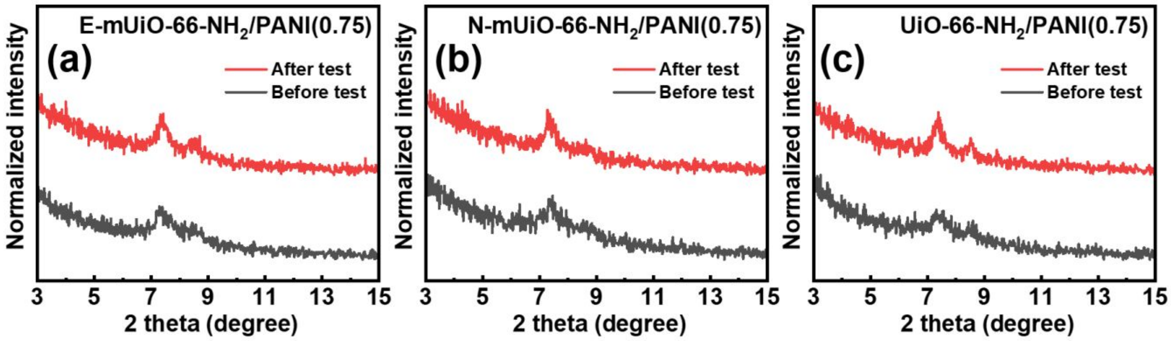

Figure S7. GIXRD patterns of thin films of (a) E-mUiO-66-NH_2/PANI(0.75), (b) N-mUiO-66-NH_2/PANI(0.75) and (c) UiO-66-NH_2/PANI(0.75), collected before and after 50 cycles of CV scan in 1.0 M HCl (aq). Each CV experiment was conducted between 0 and +0.8 V *vs*. Ag/AgCl/NaCl (3 M) at a scan rate of 50 mV/s.

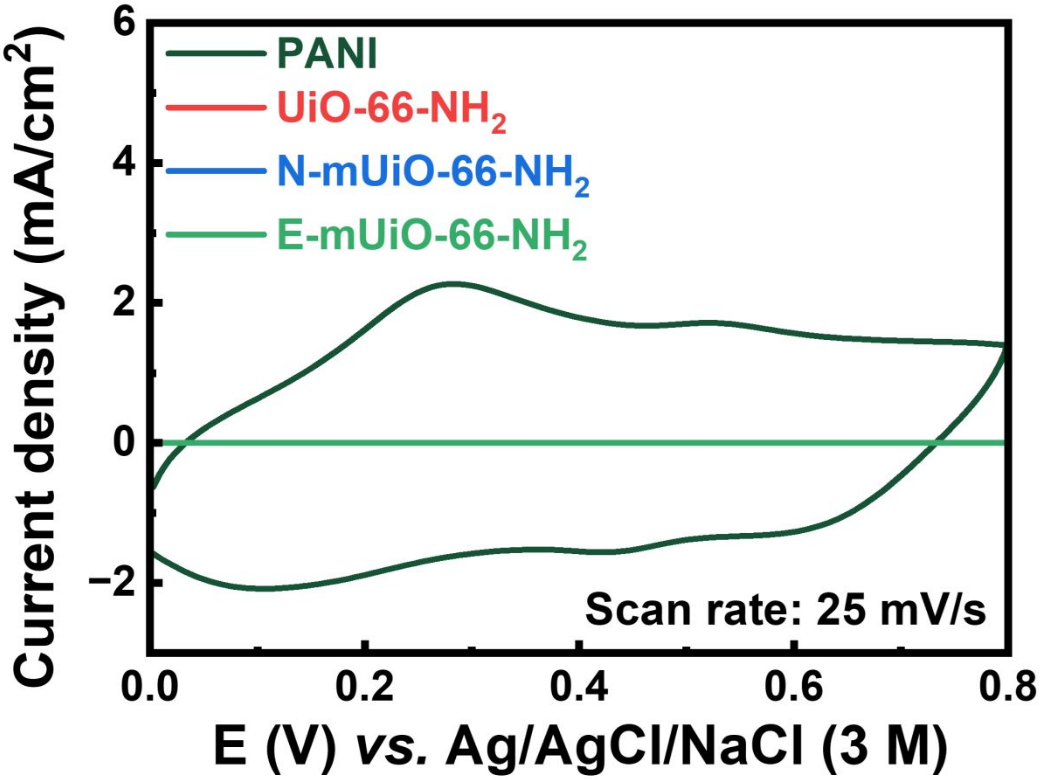

Figure S8. CV curves of modified electrodes with PANI, UiO-66-NH_2, N-mUiO-66-NH_2 and E-mUiO-66-NH_2, measured at 25 mV/s in 1.0 M HCl (aq).

Table S3. Mass loading of PANI in each PANI-based modified electrode.

Materials	PANI (wt%) (From **Table S1**)	Thin-film loading (mg/cm^2)	PANI loading (mg/cm^2)
PANI	100	0.173	0.173
E-mUiO-66-NH_2/PANI(0.25)	77.2	0.173	0.134
E-mUiO-66-NH_2/PANI(0.5)	54.7	0.173	0.095
E-mUiO-66-NH_2/PANI(0.75)	42.7	0.173	0.074
E-mUiO-66-NH_2/PANI(1)	36.9	0.173	0.064
N-mUiO-66-NH_2/PANI(0.75)	44.4	0.173	0.077
UiO-66-NH_2/PANI(0.75)	48.3	0.173	0.084

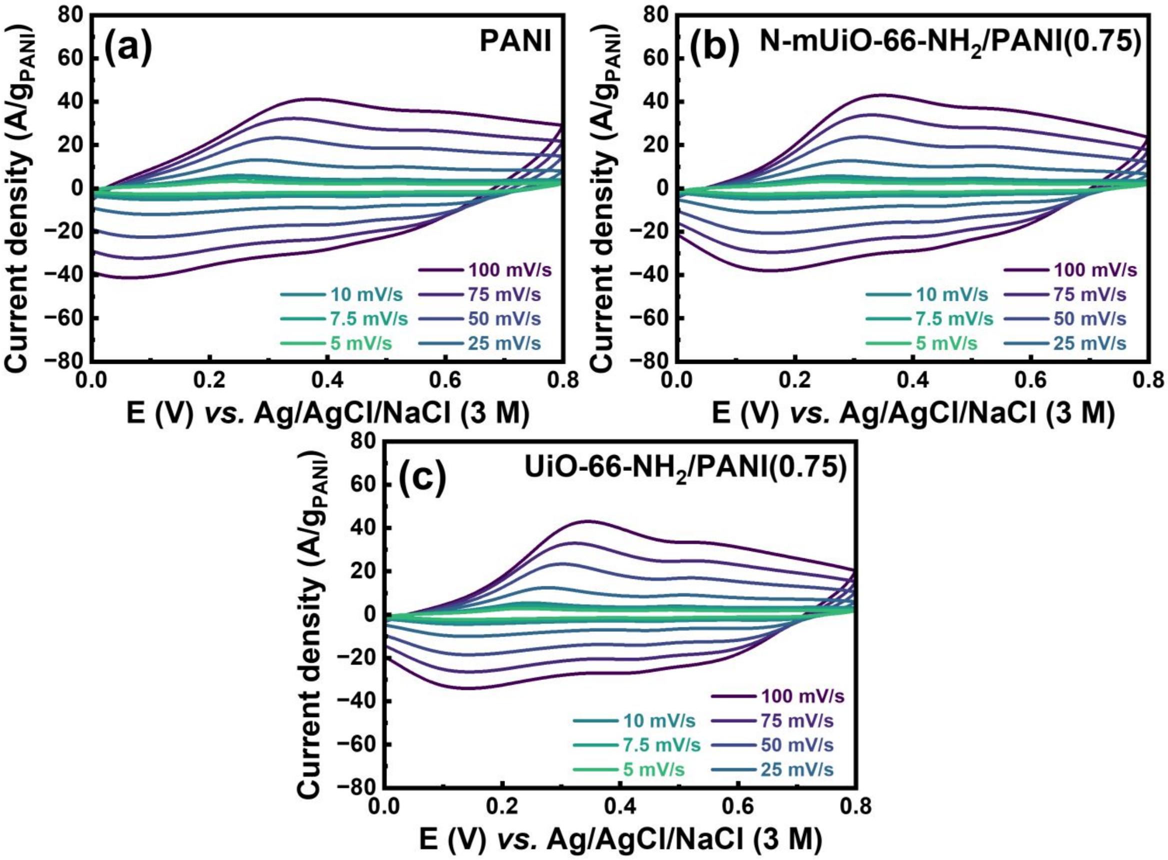

Figure S9. CV curves of modified electrodes with (a) PANI, (b) N-mUiO-66-NH_2/PANI(0.75) and (c) UiO-66-NH_2/PANI(0.75), measured at various scan rates in 1.0 M HCl (aq).

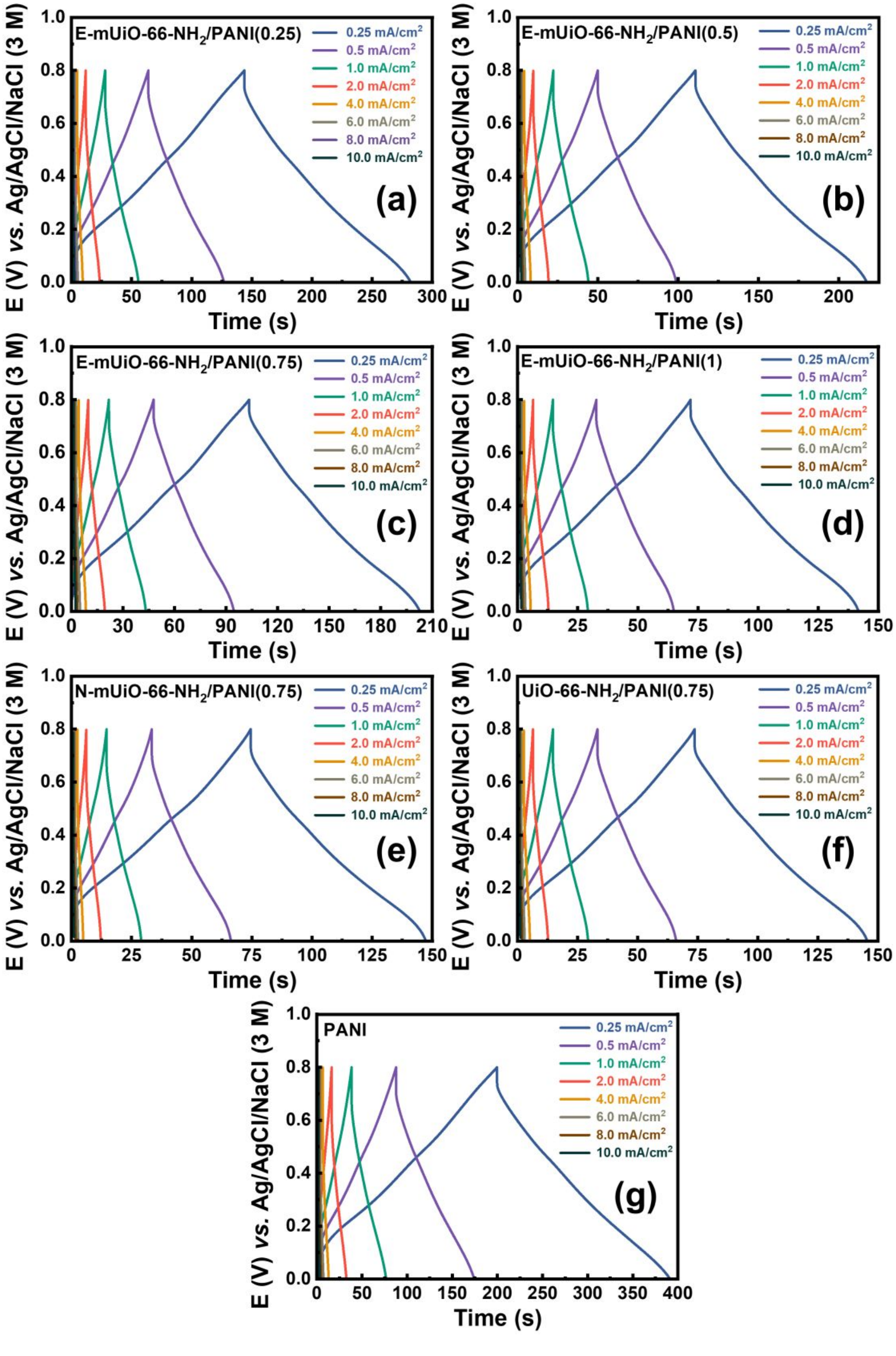

Figure S10. GCD curves of modified electrodes with (a) E-mUiO-66-NH_2/PANI(0.25), (b) E-mUiO-66-NH_2/PANI(0.5), (c) E-mUiO-66-NH_2/PANI(0.75), (d) E-mUiO-66-NH_2/PANI(1), (e) N-mUiO-66-NH_2/PANI(0.75), (f) UiO-66-NH_2/PANI(0.75) and (g) PANI, collected at various charge-discharge rates in 1.0 M HCl (aq).

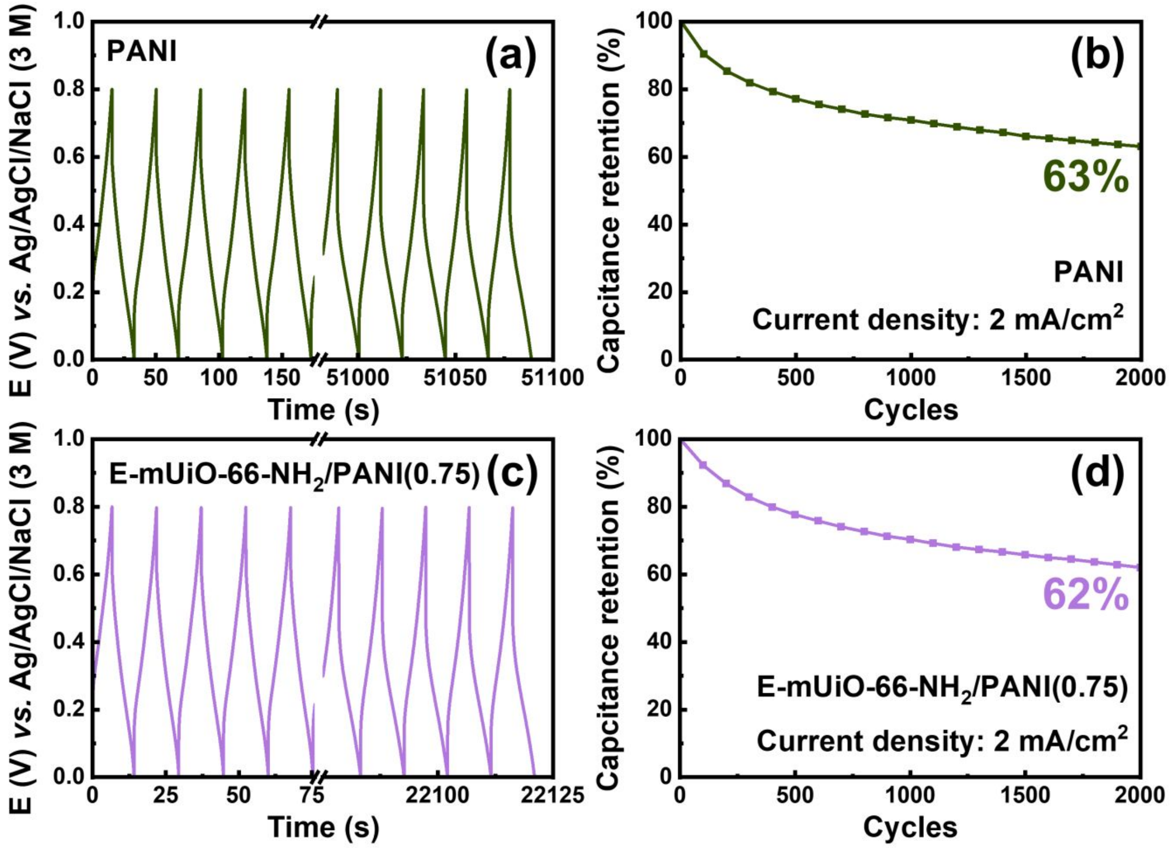

Figure S11. GCD curves of modified electrodes with (a) PANI and (c) E-mUiO-66-NH_2/PANI(0.75), measured at 2 mA/cm^2 for 2000 cycles in 1.0 M HCl (aq). Panels (b) and (d) show the capacitance retentions calculated from the data in (a) and (c), respectively.

Table S4. A partial list of PANI reported for supercapacitors and their performances, sorted based on specific capacitance.

Electrode Material	Specific capacitance (F/g)	Charge-discharge rate	Electrolyte	Method / Potential window (V)	Reference
PANI/UiO-66	1015	1 A/g	1 M H_2SO_4	GCD/0.8	[5]
PANI nanowires	818	1 mA/cm^2	1 M H_2SO_4	GCD/0.7	[6]
Mesoporous PANI	532	1.5 A/g	1 M H_2SO_4	GCD/0.8	[7]
PANI@ZrBTB-SO_3(1:1.5)	515	0.5 mA/cm^2	0.2 M HCl	GCD/0.8	[8]
Graphene/PANI nanofibers	480	0.1 A/g	2 M H_2SO_4	GCD/1.0	[9]
E-mUiO-66-NH_2/PANI(0.75)	421	0.25 mA/cm^2	1 M HCl	GCD/0.8	This work
ZIF-8/PANI	395	0.2 A/g	1 M H_2SO_4	GCD/1.0	[10]
PPH [a]	388	20 mA/cm^2	1 M H_2SO_4	GCD/0.8	[11]
PANI/Fe-BTC	388	20 mV/s	0.5 M H_2SO_4	CV/0.9	[12]
PANI@MIL-101	378	1 mV/s	1 M H_2SO_4	CV/1.0	[13]
PANI-ZIF-67-CC [b]	371	10 mV/s	3 M KCl	CV/1.2	[14]
Porous PANI	361	0.25 A/g	0.5 M H_2SO_4	GCD/0.8	[15]
PANI/Fe_3O_4	214	1 mA/cm^2	1 M H_2SO_4	GCD/0.75	[16]
PANI-$LiPF_6$	80	2 mA/cm^2	Polymer electrolyte	GCD/1.0	[17]
PANI-UiO-66-NH_2	79.8	5 mV/s	0.01 M HCl	CV/0.6	[18]

[a] PPH = porous polyaniline hydrogel
[b] CC = carbon cloth

References:

1. Ho, W. H.; Li, S.-C.; Wang, Y.-C.; Chang, T.-E.; Chiang, Y.-T.; Li, Y.-P.; Kung, C.-W., Proton-Conductive Cerium-Based Metal–Organic Frameworks. *ACS Appl. Mater. Interfaces* **2021,** *13*, 55358-55366.

2. Shen, C.-H.; Chuang, C.-H.; Gu, Y.-J.; Ho, W. H.; Song, Y.-D.; Chen, Y.-C.; Wang, Y.-C.; Kung, C.-W., Cerium-Based Metal–Organic Framework Nanocrystals Interconnected by Carbon Nanotubes for Boosting Electrochemical Capacitor Performance. *ACS Appl. Mater. Interfaces* **2021,** *13*, 16418-16426.

3. Song, Y.-D.; Ho, W. H.; Chen, Y.-C.; Li, J.-H.; Wang, Y.-S.; Gu, Y.-J.; Chuang, C.-H.; Kung, C.-W., Selective Formation of Polyaniline Confined in the Nanopores of a Metal–Organic Framework for Supercapacitors. *Chem. Eur. J.* **2021,** *27*, 3560-3567.

4. Wang, Y.-C.; Yen, J.-H.; Huang, C.-W.; Chang, T.-E.; Chen, Y.-L.; Chen, Y.-H.; Lin, C.-Y.; Kung, C.-W., Metal–Organic Framework-Derived Electrocatalysts Competent for the Conversion of Acrylonitrile to Adiponitrile. *ACS Appl. Mater. Interfaces* **2022,** *14*, 35534-35544.

5. Shao, L.; Wang, Q.; Ma, Z.; Ji, Z.; Wang, X.; Song, D.; Liu, Y.; Wang, N., A High-Capacitance Flexible Solid-State Supercapacitor Based on Polyaniline and Metal-Organic Framework (Uio-66) Composites. *J. Power Sources* **2018,** *379*, 350-361.

6. Gupta, V.; Miura, N., Electrochemically Deposited Polyaniline Nanowire's Network: A High-Performance Electrode Material for Redox Supercapacitor. *Electrochem. Solid-State Lett.* **2005,** *8*, A630.

7. Khdary, N. H.; Abdesalam, M. E.; Enany, G. E., Mesoporous Polyaniline Films for High Performance Supercapacitors. *J. Electrochem. Soc.* **2014,** *161*, G63.

8. Tsai, M.-D.; Chen, Y.-L.; Chang, J.-W.; Yang, S.-C.; Kung, C.-W., Sulfonate-Functionalized Two-Dimensional Metal–Organic Framework as a "Dispersant" for Polyaniline to Boost Its Electrochemical Capacitive Performance. *ACS Appl. Energy Mater.* **2023,** *6*, 11268-11277.

9. Zhang, K.; Zhang, L. L.; Zhao, X.; Wu, J., Graphene/Polyaniline Nanofiber Composites as Supercapacitor Electrodes. *Chem. Mat.* **2010,** *22*, 1392-1401.

10. Udayan, A. P. M.; Sadak, O.; Gunasekaran, S., Metal–Organic Framework/Polyaniline Nanocomposites for Lightweight Energy Storage. *ACS Appl. Energy Mater.* **2020,** *3*, 12368-12377.

11. Li, L.; Ai, Z.; Wu, J., A Robust Polyaniline Hydrogel Electrode Enables Superior Rate Capability at Ultrahigh Mass Loadings. *Nat. Commun.* **2024,** *15*, 6591.

12. Milakin, K. A.; Gavrilov, N.; Pašti, I. A.; Morávková, Z.; Acharya, U.; Unterweger, C.; Breitenbach, S.; Zhigunov, A.; Bober, P., Polyaniline-Metal Organic Framework (Fe-BTC) Composite for Electrochemical Applications. *Polymer* **2020,** *208*, 122945.

13. Aliev, S. B.; Samsonenko, D. G.; Maksimovskiy, E. A.; Fedorovskaya, E. O.; Sapchenko, S. A.; Fedin, V. P., Polyaniline-Intercalated Mil-101: Selective CO_2 Sorption and Supercapacitor Properties. *New J. Chem.* **2016,** *40*, 5306-5312.

14. Wang, L.; Feng, X.; Ren, L.; Piao, Q.; Zhong, J.; Wang, Y.; Li, H.; Chen, Y.; Wang, B., Flexible Solid-State Supercapacitor Based on a Metal–Organic Framework Interwoven by Electrochemically-Deposited Pani. *J. Am. Chem. Soc.* **2015,** *137*, 4920-4923.

15. Cho, S.; Shin, K.-H.; Jang, J., Enhanced Electrochemical Performance of Highly Porous Supercapacitor Electrodes Based on Solution Processed Polyaniline Thin Films. *ACS Appl. Mater. Interfaces* **2013,** *5*, 9186-9193.

16. Radhakrishnan, S.; Prakash, S.; Rao, C. R.; Vijayan, M., Organically Soluble Bifunctional Polyaniline–Magnetite Composites for Sensing and Supercapacitor Applications. *Electrochem. Solid-State Lett.* **2009,** *12*, A84.

17. Ryu, K. S.; Kim, K. M.; Park, Y. J.; Park, N.-G.; Kang, M. G.; Chang, S. H., Redox Supercapacitor Using Polyaniline Doped with Li Salt as Electrode. *Solid State Ion.* **2002,** *152*, 861-866.

18. Milakin, K. A.; Gupta, S.; Kobera, L.; Mahun, A.; Konefał, M.; Kockova, O.; Taboubi, O.; Morávková, Z.; Chin, J. M.; Allahyarli, K., Effect of a Zr-Based Metal–Organic Framework Structure on the Properties of Its Composite with Polyaniline. *ACS Appl. Mater. Interfaces* **2023,** *15*, 23813-23823.
